# Te Ara Waiora–Implementing human papillomavirus (HPV) primary testing to prevent cervical cancer in Aotearoa New Zealand: A protocol for a non-inferiority trial

**DOI:** 10.1371/journal.pone.0280643

**Published:** 2023-03-23

**Authors:** Melanie Gibson-Helm, Tania Slater, Evelyn J. MacDonald, Kendall Stevenson, Anna Adcock, Stacie Geller, Varsha Parag, Charles Lambert, Matthew Bennett, Merilyn Hibma, Peter Sykes, Marion Saville, David Hawkes, Jo-Ann Stanton, Mary-Ann Clueard, Grahame Jelley, Bev Lawton

**Affiliations:** 1 Te Tātai Hauora o Hine–National Centre for Women’s Health Research Aotearoa (NCWHRA), Victoria University of Wellington, Wellington, New Zealand; 2 National Center of Excellence in Women’s Health, University of Illinois, Chicago, IL, United States of America; 3 National Institute for Health Innovation, University of Auckland, Auckland, New Zealand; 4 Department of Pathology, University of Otago, Dunedin, New Zealand; 5 Christchurch Hospital and University of Otago, Canterbury, New Zealand; 6 Australian Centre for the Prevention of Cervical Cancer, Melbourne, Australia; 7 Mahitahi Hauora Primary Health Entity, Northland, New Zealand; PLOS: Public Library of Science, UNITED KINGDOM

## Abstract

**Background:**

Cervical cancer is caused by high-risk types of human papillomavirus (HPV). Testing for high-risk HPV is a more sensitive screening method than cervical cytology for detecting cervical changes that may lead to cancer. Consistent with recent evidence of efficacy and acceptability, Aotearoa New Zealand plans to introduce HPV testing as the primary approach to screening, replacing cervical cytology, from mid-2023. Any equitable cervical screening programme must be effective across a diverse population, including women that the current programme fails to reach, particularly Māori and those in rural areas. Currently, we do not know the best model for implementing an equitable HPV self-testing screening programme.

**Methods:**

This implementation trial aims to assess whether a universal offer of HPV self-testing (offered to all people eligible for cervical screening) achieves non-inferior screening coverage (equal) to a universal offer of cervical cytology alone (the present programme). The study population is all people aged from 24.5 to 70 years due for cervical screening in a 12-month period (including those whose screening is overdue or who have never had screening). A range of quantitative and qualitative secondary outcomes will be explored, including barriers and facilitators across screening and diagnostic pathways. This study takes place in Te Tai Tokerau/Northland which covers a diverse range of urban and rural areas and has a large Indigenous Māori population. A total of fourteen practices will be involved. Seven practices will offer HPV self-testing universally to approximately 2800 women and will be compared to seven practices providing routine clinical care (offer of cervical cytology) to an approximately equal number of women.

**Discussion:**

This trial will answer important questions about how to implement an equitable, high-quality, effective national programme offering HPV self-testing as the primary screening method for cervical cancer prevention.

**Trial registration:**

Prospectively registered with the Australian New Zealand Clinical Trials Registry 07/12/2021: ACTRN12621001675819.

## Introduction

### Background and rationale

Cervical cancer is now preventable by vaccination and screening [1]. Screening can detect pre-cancerous lesions that can be treated, thereby preventing the development of potentially fatal disease. Cervical cancer is caused by high-risk types of human papillomavirus (HPV). It is globally recognised that testing for high-risk HPV is more sensitive than cervical cytology for detecting cervical changes that may lead to cancer [2, 3]. Testing for high-risk HPV as a primary screening method is now the gold standard for cervical cancer prevention [4]. In HPV *self-testing*, the sample used is a *self-collected* vaginal swab, which is then analysed by a laboratory. Self-testing is as effective as a clinician-collected sample, when the testing platform uses polymerase chain reaction [5]. Importantly, HPV self-testing is acceptable and accessible to Māori, the Indigenous people of Aotearoa New Zealand, who have a higher burden of cervical cancer [6–8]. Additionally, primary healthcare providers find HPV self-testing to be feasible in their practices [6–8].

Cervical cytology is the primary screening method in Aotearoa New Zealand. Switching to HPV testing as the primary screen for cervical cancer prevention has the potential to reduce incident cervical cancers by 15% annually [9, 10]. The National Cervical Screening Programme (NCSP) was established in 1990 to reduce cervical cancer incidence and death. The NCSP uses a register and three-yearly screening and recall systems, administered centrally and through primary care. Through routine, regular screening, the NCSP aims to detect precancerous cell changes, which, if treated, prevents cancer. Since the introduction of the NCSP, the incidence of cervical cancer has fallen by approximately 50% [11]; however, approximately 186 women are still diagnosed with invasive cervical cancer each year and, in 2018, 69 women died of cervical cancer [12]. Of the women diagnosed with cervical cancer in New Zealand, around 88% have not received adequate screening [13].

The current NCSP has significant inequities: 41% of Māori women are under-screened or never-screened compared to 21% of European/other women [14]. These inequities have grown during the COVID-19 pandemic [15]. Cervical cancer detection programmes require high levels of screening to be successful in reducing cancer rates [16]. Similar to other Indigenous peoples [17], Māori women are approximately twice as likely to be diagnosed with cervical cancer than non-Māori women and 2.5 times more likely to die of cervical cancer [18].

The present NCSP involves a speculum exam and taking a cervical sample for cytology, an invasive procedure that requires attending a clinic. For Māori women, barriers to screening include having to attend a clinic, rurality, cost, no transport or time off work, fear of results, and the pain, embarrassment, or discomfort of a genital examination [7]. Self-testing for HPV overcomes access issues for Māori and for other under-screened groups [6, 8].

A new NCSP offering HPV primary testing is planned to be introduced in mid-2023. Aotearoa New Zealand has the opportunity to be one of the first high income countries to switch to HPV self-testing as the primary screening method for cervical cancer prevention.

For the new NCSP to be effective, it must be accessible and acceptable to women that the current NCSP fails to reach, particularly Māori and those in rural areas. Such a programme also needs to ensure that any post-screening triage system does not create barriers to diagnostic care [19]. Implementation research, based in communities who are currently underserved, is required to understand how to deliver an equitable NCSP.

This study will inform how to implement an equitable, high-quality NCSP, utilising a universal offer of HPV self-testing.

### Objectives

This implementation trial aims to assess whether a universal offer of HPV self-testing (offered to all people eligible for cervical screening) achieves non-inferior screening coverage (equal) to a universal offer of cervical cytology alone (the present NCSP).

We hypothesise that a universal offer of HPV self-testing leads to at least as many women having cervical screening as standard care (universal offer of cervical cytology only).

A concurrent qualitative component will explore barriers and facilitators for women accessing the triage system (to cytology or colposcopy) following a positive high-risk HPV result. Stakeholder views on delivering a universal offer of HPV self-testing in primary care will also be sought. Additionally, barriers and facilitators will be investigated for 25-year-old women joining the NCSP, and for women who are not enrolled in primary care.

### Trial design

**Fig 1:**
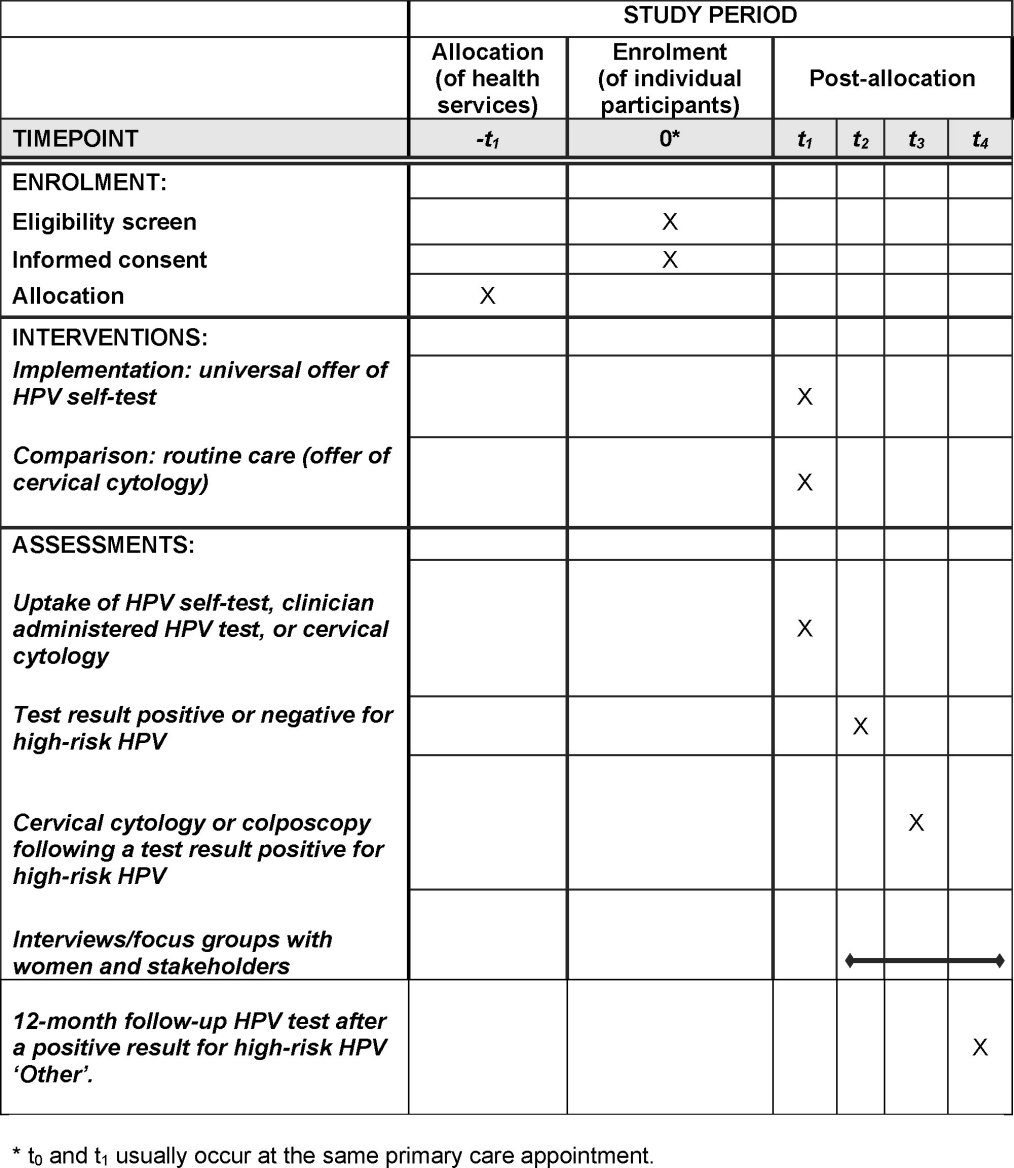
Te Ara Waiora–Implementing human papillomavirus (HPV) primary testing in Aotearoa New Zealand: Schedule of enrolment, interventions, and assessments.

**Fig 2:**
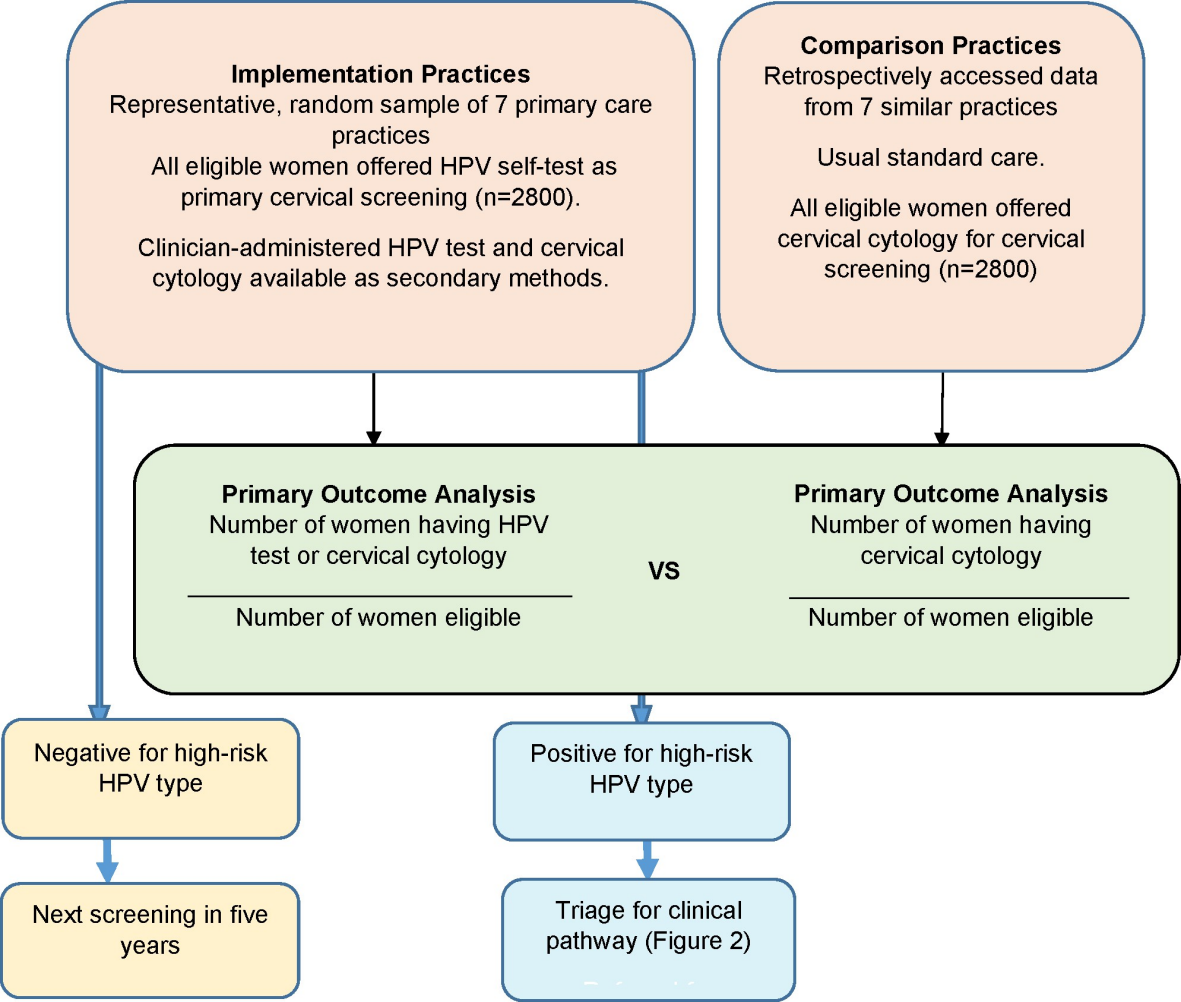
Te Ara Waiora–Implementing human papillomavirus (HPV) primary testing in Aotearoa New Zealand: Study design.

This non-inferiority, implementation trial aims to assess whether a universal offer of HPV self-testing achieves non-inferior screening coverage to a universal offer of cervical cytology alone. The trial schedule is summarised in Fig 1 and the trial design is summarised in Fig 2.

## Methods: Participants, interventions, and outcomes

### Study setting

Te Tai Tokerau—Northland covers a diverse range of urban and rural areas and has a large Māori population. Approximately 34% of the total Northland population of 175,400 identify as Māori [20]. Māori women in Northland have almost twice the incidence of cervical cancer and almost four times the mortality compared to non-Māori [21]. Prior to the COVID-19 pandemic, cervical screening coverage for Māori women in Te Tai Tokerau was approximately 55%[14].

Primary health organisations (PHO) ensure the provision of essential primary health care services, mostly through general practices, to people who are enrolled with the PHO. There are approximately 30 PHO in Aotearoa New Zealand and they care for almost all New Zealanders [22]. Mahitahi Hauora is Te Tai Tokerau’s Primary Health Entity (PHE) formed through consolidation of several PHOs. Mahitahi Hauora is a collaborative endeavour with support from the Northland regional hospital, Māori-led health providers, general practice, and iwi leaders. This implementation trial is a partnership between Te Tātai Hauora o Hine–National Centre for Women’s Health Research Aotearoa (NCWHRA) and Mahitahi Hauora. Extensive consultation contributed to the design of this research and subsequent funding acquisition. Consultation was with research kaumātua (Māori community leaders), general practitioners, Mahitahi Hauora, community, and the colposcopy service of the Northland regional hospital.

### Outcomes

#### Primary outcome

The primary outcome is cervical screening coverage over a one-year period. At implementation practices this is measured as the proportion of the eligible population in the study period who are screened by HPV self-test, clinician-administered HPV test or cervical cytology. At comparison practices this is measured as the proportion of the eligible population in the study period who are screened by cervical cytology alone.

#### Secondary outcomes

The following outcomes will be collected from the implementation practices (offering HPV self-testing):

1. Proportion of women having an HPV self-test.
2. Proportion of women having either an HPV self-test, clinician-administered HPV test or cervical cytology (uptake of screening).
3. Proportion of women with a positive result for high-risk HPV (HPV16, HPV18 or HPV ‘Other’).
4. Proportion of women who have cervical cytology following a positive result for HPV ‘Other’.
5. Proportion of women who return for an HPV test after one year following a positive result for HPV ‘Other’ and a negative/mild dysplasia cytology result.
6. Proportion of women attending colposcopy following a positive result for HPV16 or HPV18.
7. Prevalence of high-grade lesions (CIN2, CIN3, carcinoma) among women attending colposcopy.

These outcomes will be stratified by, or adjusted for, ethnicity, deprivation index and screening history to understand screening uptake and post-screening care pathways in these groups.

#### Qualitative outcomes

1. Barriers and facilitators to the triage system for women with a positive test result for high-risk HPV.
2. Barriers and facilitators to delivering HPV self-testing in primary care.
3. Barriers and facilitators to women beginning screening at 25 years of age.
4. Barriers and facilitators to accessing screening, for women who are not enrolled in primary care.

These outcomes will help inform how the NCSP can facilitate access to screening and how to ensure an equitable triage system for women with positive test results for high-risk HPV.

### Sample size

The NCSP coverage report from December 2019 for Northland shows that approximately 72.5% of women (33,403/46,090) aged 25–69 were screened over a period of three years [15]. A total of fourteen practices and 5600 women (seven practices and 2800 women in each group) will give 95% power at a one-sided significance level of 2.5% to detect a non-inferiority margin of 10% in cervical screening coverage (primary outcome) between the two groups. This margin of error was chosen as it is the smallest value that is clinically relevant. For this sample size a screening rate over a one-year period of 72.5% in the comparative data and 72.5% in the implementation practices was assumed, and an average practice size of 400 participants and intra-cluster correlation coefficient of 0.01.

### Implementation practice eligibility criteria and consent

Seven practices were randomly selected from the 22 practices that have a moderate to high proportion of Māori (≥ 15% of the practice’s population). This random selection is stratified by practice size and rurality to ensure representation from small-rural, small-urban, large-rural, and large-urban practices. These seven practices (implementation practices) will offer HPV self-testing to all eligible women over a 12-month period (approximately 2800 women). Consent to participate in the implementation was sought from each practice.

### Explanation for the choice of comparators, comparison practice eligibility criteria and consent

Seven comparison primary care practices in Te Tai Tokerau with similar characteristics to the implementation practices have been selected (practice population size, rurality, ≥15% Māori). These practices will provide standard care (cervical cytology screening) during the same 12-month implementation period. Retrospective, de-identified cervical screening data will be requested for all women in the eligible population (approximately 2800 women) who have cervical cytology or have no cervical screening during the study period. This information is required to calculate the primary outcome: screening coverage. Informed consent will not be sought from individual women. Any changes to standard care during the study period will be documented.

### Assignment of interventions: Allocation

#### Sequence generation, concealment mechanism, implementation of allocation

Practices were randomly selected from the 22 eligible practices that have a moderate to high proportion of Māori, using computer generated random numbers. Randomisation was stratified by practice size and rurality. The randomisation sequence was generated by the trial statistician, blinded to the identity or location of the eligible practices. Researchers working directly with Mahitahi Hauora had no role in the allocation process. The trial statistician assigned the practices based on the randomisation sequence and provided the list of allocated practice numbers to the researchers working with Mahitahi Hauora, who then identified the practices.

#### Blinding

Blinding of participants and clinicians is not possible. One trial statistician is unblinded and compiles the dataset, while a second trial statistician is blinded and conducts the primary outcome analysis. Secondary outcomes are only assessed in the implementation group; hence blinding is not possible.

### Intervention description

#### Recruitment of participants

Implementation practices will deliver a universal offer of HPV self-testing. This means that practice staff will offer an HPV self-test as the primary screening test to all eligible woman due or overdue for cervical screening. If the woman declines the HPV self-test, she will be offered conventional cervical cytology screening. Posters and flyers with self-test information are displayed in these practices.

#### Participant inclusion criteria

The NCSP eligibility policy is that “anyone with a cervix or vagina who has ever been sexually active should be offered three-yearly cervical screening from age 25 to age 69.” For this study, people eligible for cervical screening in the 12-month study period, attending an implementation practice, and aged from 24.5 to 70.0 years are eligible to participate. This includes women who are due for screening (including up to six months before their screening due date), overdue for screening (four years or more since their last screening i.e. one year or more overdue) or who have never had screening.

Symptoms raising the possibility of cervical pathology require investigation and diagnosis. Symptoms include post-coital bleeding, unexplained intermenstrual bleeding, any post-menopausal bleeding, unexplained, persistent, and unusual vaginal discharge, or unexplained persistent deep dyspareunia. Women meeting the eligibility criteria but who have symptoms may still have an HPV test.

#### Participant exclusion criteria

Women who have had treatment for cervical abnormalities and are under active follow up (e.g. still undergoing test of cure) are not eligible. A test of cure is HPV testing and cytology (co-testing) on two occasions, 12 months apart. Negative results for both tests are required twice before the test of cure is complete. Women who have had treatment for cervical abnormalities in the past and are *overdue* for test of cure are eligible.

#### Participant informed consent

General practice staff, led by a key clinician (champion) at each practice, undertake a clinical update and train-the-trainer programme, provided by expert members of the research team. In-person training delivery is preferred, but due to COVID-19 restrictions, training can also be by zoom or pre-recorded video. Training is augmented by ongoing contact between the research team, champions, and staff. Trained clinicians or kaiāwhina (community health or outreach worker) obtain informed consent from women for a copy of HPV results (and if colposcopy is required, copies of histology results) to be sent to the researchers as well as the ordering health practitioner.

#### Additional participant consent provisions for collection and use of data

The consent form includes the option to give permission to be contacted by a qualitative researcher about participating in an interview on their experiences of the HPV self-test and any subsequent follow-up. Key stakeholders including general practice and regional hospital colposcopy staff, whānau (family) and community representatives will be identified by Mahitahi Hauora, the steering committee and the research team, and will be invited to participate in an interview.

#### Taking the HPV test

Women are at the centre of this study, and we are led by their preferences. Previous research suggests that the majority of women will self-collect the sample, although they may choose to have the sample collected by a clinician [6]. Women receive verbal, written and/or pictorial instructions from a trained clinician or kaiāwhina about how to collect a vaginal sample using a nylon-flocked swab (Copan FLOQswab, Copan, Italy). Most sample collection will take place in the practice, but some women self-collect the sample in their homes during a kaiāwhina visit.

#### Laboratory test and process

Samples are transported at room temperature to the diagnostic laboratory as part of the practice’s routine laboratory collection. HPV genotyping is carried out using Abbott HPV assays, which are able to give partial genotyping results to identify HPV16, HPV18, and HPV ‘Other’ (a combined result for the presence of 12 other high-risk HPV types: 31, 33, 35, 39, 45, 51, 52, 56, 58, 59, 66, 68) [23]. The use of dry vaginal swabs has been validated in international published studies [24, 25] and the laboratory is undertaking this testing ‘off-label’ as there were no PCR-based HPV assays with a manufacturer claim for use with self-collected samples at the beginning of this study. The samples are disposed of in line with standard pathology laboratory procedures.

#### Results of HPV tests

Test results are sent electronically (encrypted as other routine results) to the ordering primary care clinician and entered in the practice patient management system, as cytology results would be. The practice sends reminders to women as they would for cervical cytology recall. Results are also automatically sent encrypted to the canSCREEN^®^ registry platform [26] (described in detail later), and to the principal investigator (BL). If the test result is ‘unsatisfactory’ the lab requests another sample.

#### Returning results

Women choose the method by which they receive their HPV test result from the clinician. This might be by text, if negative, or by phone or in person at the practice. Results that are positive for high-risk HPV are given to women in person or by telephone by the ordering clinician in a sensitive and appropriate way. Women may choose to have support alongside them when receiving the results. It is emphasised by the clinician that a positive test result for high-risk HPV does not mean that the woman has cancer, but it may indicate pre-cancer changes on the cervix that can be identified and treated. Women receive information about the referral process to colposcopy and the examination process itself. Women and whānau can be navigated through these steps and supported to colposcopy through existing support networks where available.

#### Triage

It is the responsibility of the ordering clinician to organise the referral for colposcopy for women with positive test results for high-risk HPV. A standardised pro-forma letter is sent to the colposcopy department at the regional hospital including usual clinical referral information and stating that the woman is part of this study.

**Fig 3:**
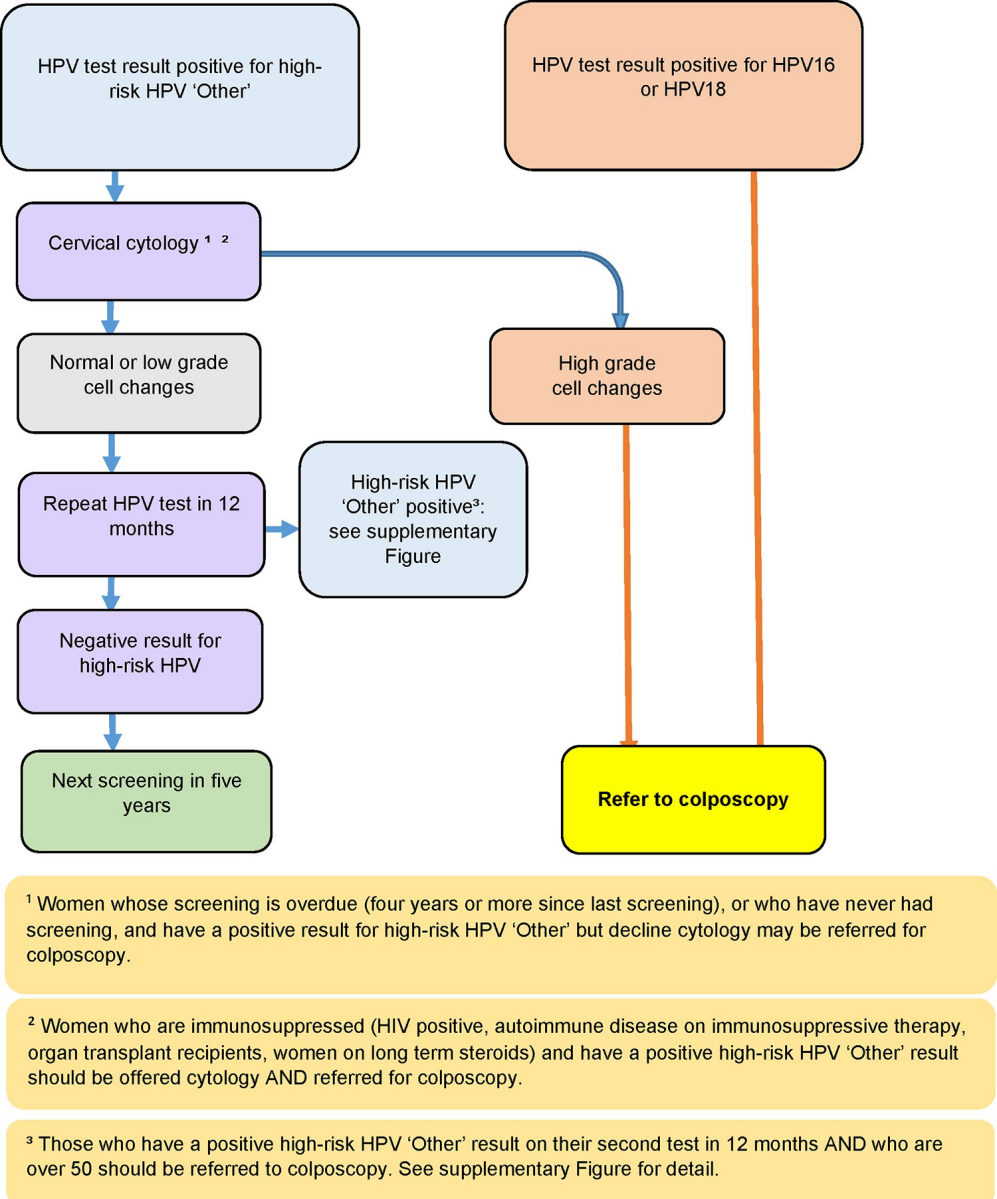
Te Ara Waiora–Implementing human papillomavirus (HPV) primary testing in Aotearoa New Zealand: Triage and referral pathways following an HPV test.

**Fig 4:**
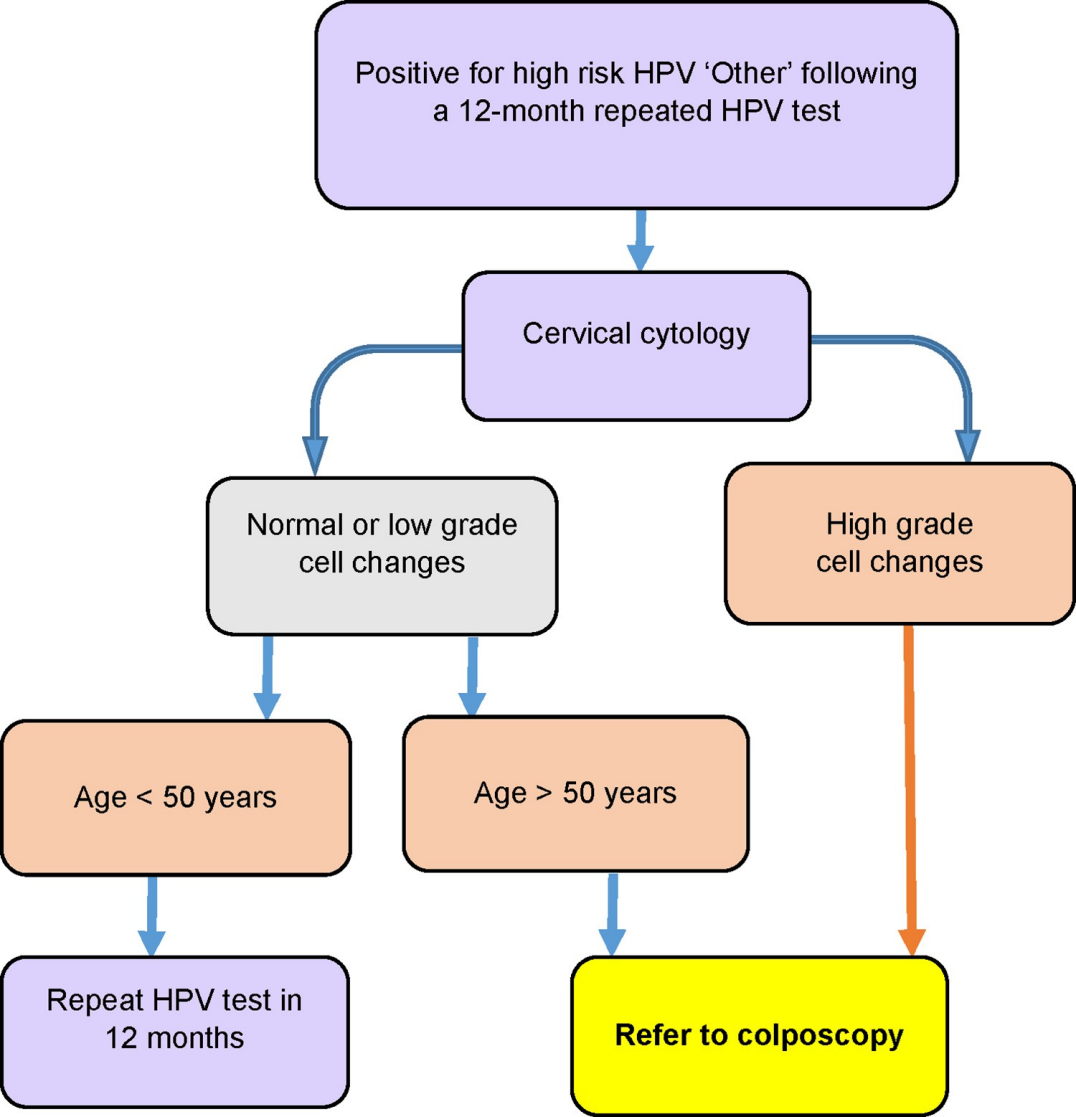
Te Ara Waiora–Implementing human papillomavirus (HPV) primary testing in Aotearoa New Zealand: Triage and referral pathway for women who have two positive test results for HPV ‘other’ 12 months apart.

Referring all women with positive test results for high-risk HPV straight to colposcopy would increase the number of unnecessary colposcopies, potentially overwhelming the secondary service. To mitigate this, a triage system has been co-developed between the researchers, the colposcopy service at the regional hospital, and the Ministry of Health (Figs 3 and 4). There are unique questions about triaging that will be answered through this study’s qualitative research component, especially about triaging women with positive test results for HPV ‘Other’ to cytology.

**Strategies to improve adherence to intervention.** The practices coordinate the testing, community outreach, results and follow up. Mahitahi Hauora are involved at all levels of the study. Mahitahi Hauora have ownership of their data and all results. This partnership is valued and there are many opportunities for learning from each other. The project includes local workforce development with named and funded investigators from Mahitahi Hauora involved in governance and advisory roles. Researchers are readily available to support implementation practices, make regular site visits, and provide ongoing training.

#### Plans to promote participant retention and complete follow-up

The ordering clinician and the practices have the primary responsibility for follow up. The researchers support the practices by using canSCREEN^®^ as a clinical safety net, to identify and contact women who have not presented for follow-up tests and/or colposcopy appointments. Follow up correspondence is generated by canSCREEN^®^ and sent by text or post to the woman. The canSCREEN^®^ platform will generate a reminder for women who test negative for high-risk HPV that their next screening test is due in five years.

#### Provisions for post-trial care

Follow up colposcopy and treatment performed within the public health service are free for New Zealand citizens and permanent residents. At the conclusion of the trial, it is anticipated that data contained within canSCREEN^®^ will be migrated to the new national register to support women’s ongoing participation in cervical screening.

### Data collection and management

#### Plans for assessment and collection of outcomes, data management and confidentiality

Data management plans for both implementation and comparison practices were developed, reviewed by the relevant human research ethics committee (see ethics approval section for full details), and are described here.

The study will link quantitative data extracted from the following sources:

- canSCREEN^®^
- Participant medical records
- The National Screening Unit (NSU)
- Ministry of Health National Health Index (NHI)
- The National Immunisation Register (NIR)

#### canSCREEN^®^

The canSCREEN^®^ platform is a fully developed cancer screening registry platform developed and operated by Australian Centre for the Prevention of Cervical Cancer (ACPCC) [26]. It was customised to meet the needs of Te Tai Tokerau/Northland and perform the equivalent function as the national register for women who have an HPV test. This was necessary because the current NCSP register cannot incorporate HPV results, act on results, perform follow up or recall. The canSCREEN^®^ platform holds information about HPV testing and follow-up: practice attended, HPV test results, cytology triage (for HPV ‘Other’), colposcopy referral, attendance, and histology, and treatment/ discharge/ 12-month follow-up. It also holds information relevant to monitoring eligibility and safety such as name, address, and date of birth.

The HPV test results are transferred electronically from the laboratory directly into canSCREEN^®^. The colposcopy assessment data is entered in canSCREEN^®^ by the team leader of the colposcopy department at Northland regional hospital. Data entry training is provided by the canSCREEN^®^ team.

The canSCREEN^®^ platform is highly secure and supports role-based access by which healthcare workers and research staff can only access agreed data and functions. Data are held securely on the Microsoft^®^ Azure cloud and hosted by ACPCC. Access is only available to named investigators, clinical staff, and digital support staff. All ACPCC staff involved in this study are required to comply with strict confidentiality requirements including signing annual declarations to comply with confidentiality requests.

#### Data extracted from national datasets

Each person using a health or disability support service in Aotearoa New Zealand has a unique NHI number. Practices will provide NHI numbers of participating women to the NSU to obtain information about screening and clinical history at the start of the study period, ethnicity, Deprivation Index (socioeconomic status), and age. If there are cases where the relevant information cannot be obtained from the NSU, demographic information will be obtained from the NHI dataset held by Ministry of Health.

At the end of the implementation period, the research team will request HPV vaccination status from the NIR for participating women. This is crucial information about expected high-risk HPV positivity. Senior researchers at the NCWHRA will link NSU and NIR data to canSCREEN^®^ data. Deterministic linking will use the NHI number as the linking variable. Age and sex will be used to cross-check for any potential errors in NHI number. NHI number will be replaced with a unique study code in all research datasets for analysis.

The NSU will also be the primary source of research data for women who do not opt for an HPV self-test. The practices will provide the NHIs of the eligible population of women to the NSU (minus the women who have had an HPV test) to obtain information about cervical cytology during the implementation period, and the same demographic variables described above. The practices will remove the NHI, assign an ID number and securely transfer de-identified individual line data to NCWHRA for analysis. The practice will retain the list of NHIs and corresponding ID number.

No individuals will be identifiable in the reporting of study findings.

Study-specific source documents are maintained (for women who opt for HPV self-testing only) in locked file cabinets in locked rooms of NCWHRA. Electronic data are stored on the secure Victoria University of Wellington server, in restricted access folders and password protected files. Data will be retained for 10 years and then destroyed securely.

#### Qualitative data collection

Interviews with women will collect data about their experiences of HPV screening and further investigation after a positive high-risk HPV test result. Throughout the interviews, women will be prompted to indicate where things went well or could be improved.

Up to 10 women in each of the following triage groups will be interviewed:

- HPV16 or HPV18 test result and referral to colposcopy
- HPV ‘Other’ test result and referral to cytology
- HPV ‘Other’ test result and referral to colposcopy

These interviews will be semi-structured around six topics:

1. Their HPV self-test experience
2. Receiving their results
3. The referral process (triage to cytology or colposcopy)
4. Any support given or desired
5. Their cytology or colposcopy experience (or why they might decide not to participate in follow-up)
6. After care and re-screening information

Interviews with key stakeholders such as practice managers, doctors, nurses, kaiāwhina and PHE representatives will provide critical knowledge on barriers and facilitators to implementing HPV self-testing in primary care. These interviews will be semi-structured around three topics:

1. Their views on implementing universal HPV self-testing
2. Information or training support given or desired
3. Recommendations

Interviews/focus groups will explore enablers for 25-year-olds to participate in screening. Up to 10 interviews or a series of focus groups with 25-year-olds and their community leaders. These interviews/focus groups will be semi-structured around three topics:

1. Different screening delivery options: free screening, mail-out, self-sampling, direct approach from non-government organisations, kaiāwhina, general practice
2. Offer of free HPV vaccination
3. Communication/messaging

Interviews/focus groups, with up to 20 women and 10 kaiāwhina/kaumātua community workers/other primary care staff, will explore support needed for women who are not enrolled in primary care to participate in screening. These interviews/focus groups will be semi-structured around three topics:

1. Different screening delivery options -population based register, free screening, mailout, self-sampling, direct approach from non-government organisations, kaiāwhina
2. Support needed to access screening
3. Communication/messaging

Participants will be interviewed at a time and location that suits them. Although the research is not restricted to Māori participants, it is anticipated that many will be Māori (given the population of Te Tai Tokerau). The researchers will draw on Kaupapa Māori Research practices, which have also been described as community-up research practices, whereby whakawhanaunga (developing relationships), participant safety, and research accountability are paramount [27, 28]. Participants will sign a consent form, including permission to have their interview audio recorded and transcribed. They can stop the interview at any time. They will have the opportunity to review and approve their transcript. Transcripts will be de-identified prior to data analysis.

#### Statistical methods

Data analyses will be specified a priori in a statistical analysis plan, and analyses will be undertaken by the trial statistician using SAS version 9.4 (SAS Institute, USA). The binary outcomes will be analysed using a Linear Mixed Model with a logit link, with practice fitted as a random effect. Non-inferiority for the primary outcome will be evaluated by observing whether the lower bound of the two-sided 95% confidence intervals for the difference in screening coverage between the two groups is above the non-inferiority limit of -10. If non-inferiority is evident, assessment as to whether the implementation has effectiveness superior to that of the comparison group will be carried out using the same approach but comparing to a zero difference. The primary analyses will be carried out on an intention-to-treat basis. Per-protocol sensitivity analyses will also be conducted excluding participants who had major protocol violations. To account for any potential imbalance in underlying risk factors for screening uptake, sensitivity analyses will adjust for age, ethnicity, deprivation index, screening history and primary care practice. Analyses for the secondary outcomes will be conducted by age, ethnicity and screening status.

#### Qualitative data analysis

Interview data will be analysed thematically [29]. Transcripts will be uploaded to and coded using NVivo qualitative data analysis software (QSR International, USA). The coded data will then be organised into themes and subthemes that group participants’ talk to show commonalities and differences [29].

### Oversight and monitoring

#### Composition of the coordinating centre and trial steering committee

The study coordinating centre is NCWHRA and includes the roles of principal investigator, project manager, investigators, research assistants and kaumātua. The study coordinating centre is responsible for the day-to-day conduct of the trial and supports the practices to participate. This study is a multi-institutional, multidisciplinary, international research collaboration. Once a week, the study coordinating centre meets with Mahitahi Hauora co-investigators for an operational meeting, and the wider investigator team to discuss the conduct and progress of the trial. The study coordinating centre also meets regularly with the Northland regional hospital colposcopy service and the NSU.

#### Steering committee

In accordance with the principles under Te Tiriti o Waitangi (The Treaty of Waitangi), the steering committee works with the research partners to connect the research to communities and mitigate risk by providing subject matter and regional expert knowledge. The steering committee is chaired by Māori who live in Te Tai Tokerau and meets monthly. Membership includes research kaumātua, whānau and consumer representative(s), Mahitahi Hauora representative(s), principal investigator, project manager, local colposcopy representative(s), cervical screening representative(s).

#### Data management committee

Membership of the study’s data management committee (DMC) includes the principal investigator (general practitioner), two lead investigators (gynaecologists), the study manager, the study statisticians, and two representatives from Mahitahi Hauora (general practice services lead and data manager). The DMC is chaired by an independent researcher (public health physician). The DMC reviews recruitment progress and data completion, to confirm that the study should continue, reviews data extraction plans and dates according to recruitment progress and monitors adverse events.

#### Adverse event reporting and harms

Harm to participants in this trial is very unlikely. In that event, women are eligible to apply for compensation from the national Accident Compensation Corporation, just as if they would be if injured in an accident, at work or at home. This study uses the national Health and Disability Ethics Committee (HDEC) definitions for serious adverse events, protocol deviations, and protocol violations. Events meeting these definitions will be reported to the DMC and the HDEC, along with the actions taken in response to any event.

#### Ethics approval, plans for communicating important protocol amendments to relevant parties, consent to participate, consent for publication

National ethical approval has been granted by the Southern B HDEC (reference number 21/STH/188) with locality assessment authorisation by Northland District Health Board, Kaunihera Kaumātua, and support from Te Poutokomanawa: the Māori Health Directorate at Northland District Health Board. All major protocol amendments will be submitted to the HDEC for approval. Written informed consent will be obtained from all women undertaking an HPV test at implementation practices. Written informed consent will be obtained from all implementation and comparison practices. Model consent forms can be provided on request.

#### Timeline and trial status

**Fig 5:**
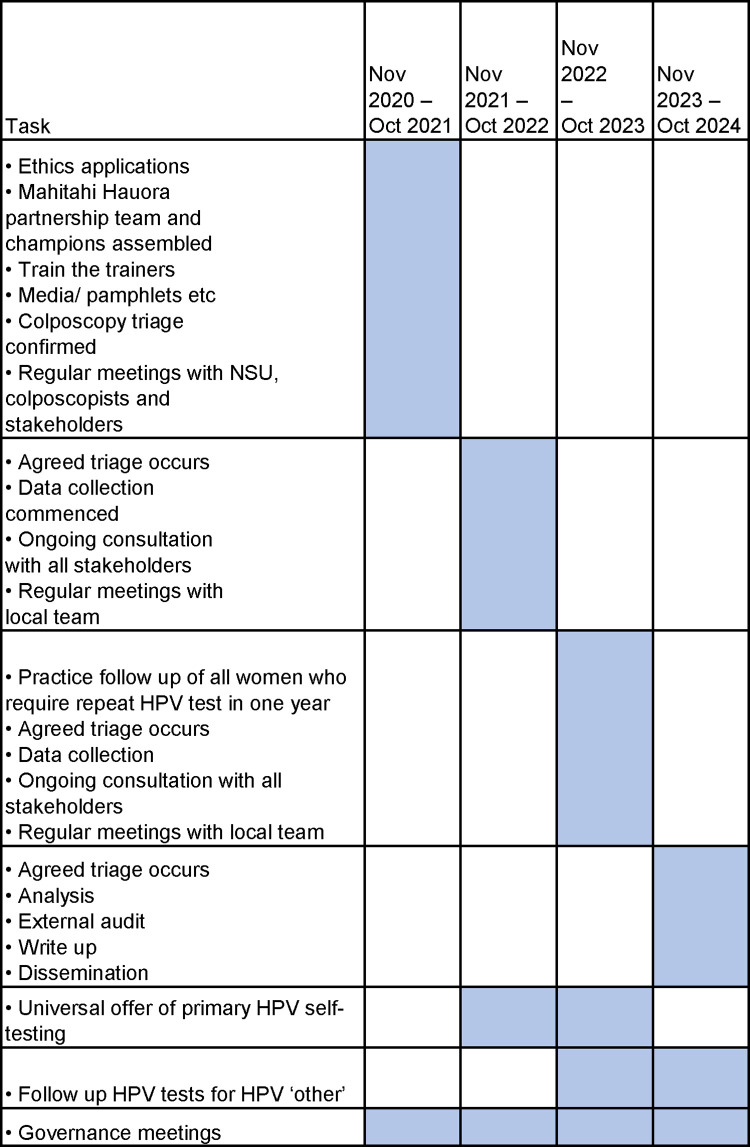
Te Ara Waiora–Implementing human papillomavirus (HPV) primary testing in Aotearoa New Zealand: Study timeline.

The first year of the project was spent preparing for the trial (Fig 5). Recruitment began in February 2022 and is expected to be completed in June 2023, with follow-up data collection to continue until October 2023. The remaining project time will be spent analysing and disseminating results. The current protocol is version four, dated October 2022.

## Discussion and dissemination plans

This study explores a model for implementing a high-quality, equitable HPV testing programme for cervical cancer prevention. Cervical screening utilising cytology has failed Māori and Indigenous populations worldwide [17]. Self-testing has the potential to improve equity and reduce cervical cancer incidence and mortality. Self-testing for high-risk HPV instead of a speculum exam can literally put screening into the hands of women.

An equitable NCSP is more critical than ever before. Cervical screening coverage has decreased over the past two years due to COVID-19 [15]. This is particularly marked in rural populations with high Māori representation [15]. Effective screening solutions involving low clinical contact such as HPV self-testing will have added national and international significance in the current pandemic era.

Aotearoa New Zealand’s new NCSP will be informed by this study in the following ways:

1. Dissemination and consultation with policy makers are priorities, to bring about policy change. Ongoing communication between the study team, general practice, NSU, Northland regional hospital, laboratory and community knits together operational questions, discussions, and resolutions in real-time. An example of this consultation is the co-developed triage system being tested in this trial for the first time in Aotearoa New Zealand (Fig 3).
2. The opportunities created by this study to hear from women and communities will inform clear and meaningful messaging about the new NCSP.
3. The priorities for the dissemination of results are firstly the community, Māori health organisations and strategic organisations that have been part of the research process. The research team also has links to multidisciplinary clinical networks across the country that are interested in using the findings from this study to change practice. Results will be disseminated to a range of national Māori health organisations such as Māori Health Provider Boards and staff hui, marae committees, Māori Women’s Welfare League, National Kaitiaki group, and Te Ora, Ngā Neehi Māori, He Hono Wahine.
4. The research team has developed, and shared, resources and study tools widely with other research teams and Indigenous researchers internationally, so all can benefit from evidence-based programme development. In addition, the findings will be published in peer reviewed journals and presented at international Indigenous forums to share and learn.

The present cytology-based NCSP has been functioning for 30 years and it is crucial that changing to an HPV primary testing NCSP is fully informed and achieved without loss of coverage and follow up. If this is maintained with the more sensitive high-risk HPV test, then the NCSP will prevent more cancers and save more lives than the present cytology program. Increasing coverage with the HPV self-test will potentially contribute to a more equitable NCSP.

The ultimate goal is to reduce cervical cancer incidence and death for Māori and non-Māori in Aotearoa New Zealand. The findings from this study will contribute to building a solid and equitable foundation for the new NCSP, with a particular focus on prioritising an NCSP that meets the needs of Māori.

## Supporting information

S1 File SPIRIT 2013 checklist: Recommended items to address in a clinical trial protocol and related documents*. (DOC)

S2 File (PDF)

## Abbreviations

ACPCC: Australian Centre for the Prevention of Cervical Cancer
CIN: Cervical intraepithelial neoplasia
DMC: Data management committee
HDEC: Health and Disability Ethics Committee
HPV: Human papillomavirus
NCSP: National Cervical Screening Programme
NCWHRA: National Centre for Women’s Health Research Aotearoa
NHI: National Health Index
NIR: National Immunisation Register
NSU: National Screening Unit
PHE: Primary Health Entity
PHO: Primary Health Organisation
