# Supplementary material for: Te Ara Waiora–Implementing human papillomavirus (HPV) primary testing to prevent cervical cancer in Aotearoa New Zealand: A protocol for a non-inferiority trial
Source: PLoS One. 2023 Mar 23;18(3):e0280643. doi: 10.1371/journal.pone.0280643 (PMC10035917; doi:10.1371/journal.pone.0280643)
Supplement: S2 File — (PDF) [file pone.0280643.s002.pdf]

NATIONAL CENTRE FOR WOMEN'S HEALTH RESEARCH AOTEAROA:
Te Tātai Hauora o Hine VICTORIA UNIVERSITY OF WELLINGTON,
PO Box 600, Wellington 6140, New Zealand
Phone +64-4-463 5497
Email NCWHRA@vuw.ac.nz
Web www.victoria.ac.nz/health

# Te Ara Waiora

# Implementing HPV primary testing to prevent cervical cancer in NZ: Te Tai Tokerau

# A non-inferiority trial

**STUDY PROTOCOL**

**October 2022**

## RESEARCH TEAM

| Investigators | Role |
|---|---|
| Professor Bev Lawton | **Principal Investigator** – Director, Te Tātai Hauora O Hine, Centre for Women's Health Research (CWHR), Faculty of Health, Victoria University of Wellington, PO Box 600, Kelburn, Wellington, 6012<br>bev.lawton@vuw.ac.nz |
| Dr E Jane MacDonald | **Co-Investigator**<br>CWHR, Victoria University of Wellington<br>e.jane.macdonald@vuw.ac.nz |
| Dr Kendall Stevenson | **Co-Investigator**<br>CWHR, Victoria University of Wellington<br>Kendall.stevenson@vuw.ac.nz |
| Anna Adcock | **Co-Investigator**<br>CWHR, Victoria University of Wellington<br>anna.adcock@vuw.ac.nz |
| Professor Stacie Geller | **Co-Investigator**<br>G. William Arends Professor of Obstetrics and Gynaecology, Director, National Center of Excellence in Women's Health, University of Illinois, Chicago.<br>sgeller@uic.edu |
| Mr Matthew Bennett | **Co-Investigator**<br>CWHR, Victoria University of Wellington<br>Matthewbe47@gmail.com |
| Mr Charlie Lambert | **Co-Investigator**<br>CWHR, Victoria University of Wellington<br>Charles.lambert@vuw.ac.nz |
| Professor Merilyn Hibma | **Co-Investigator**<br>Department of Pathology,<br>Otago University, Dunedin<br>merilyn.hibma@otago.ac.nz |
| Professor Peter Sykes | **Co-Investigator**<br>Professor of Gynaecological Oncology,<br>Christchurch Hospital and University of Otago, Canterbury<br>peter.sykes@otago.ac.nz |
| Professor Marion Saville | **Co-Investigator**<br>Executive Director, VCS Foundation, Melbourne, Australia<br>msaville@vcs.org.au |

| | |
|---|---|
| | |
| Associate Professor David Hawkes | **Co-Investigator**<br>Director, VCS Foundation, Melbourne, Australia<br>dhawkes@vcs.org.au |
| Associate Professor Jo-Anne Stanton | **Co-Investigator**<br>Department of Anatomy, University of Otago<br>J.stanton@otago.ac.nz |
| Dr Melanie Gibson | **Co-Investigator**<br>CWHR, Victoria University of Wellington<br>Melanie.gibsonhelm@vuw.ac.nz |
| Ms Varsha Parag | **Co-Investigator**<br>Lead biostatistician, National Institute for Health Innovation, University of Auckland<br>v.parag@auckland.ac.nz |
| Mrs Mary-Ann Clueard | **Co-Investigator**<br>Improvement Partner NHH, Mahitahi Hauora PH<br>Mary-Ann.Clueard@mahitahihauora.co.nz |
| Dr Grahame Jelley | **Co-Investigator**<br>Medical Director, Mahitahi Hauora PHE<br>grahame.jelley@mahitahihauora.co.nz |
| Dr Tania Slater | **Co-Investigator and Project Manager**<br>CWHR, Victoria University of Wellington<br>Tania.slater@vuw.ac.nz |


**Funding:** This project is funded by the Health Research Council (HRC 20/960) and the Ministry of Health with contribution from Mahitahi Hauora.

**Figure 1. Summary flow chart**

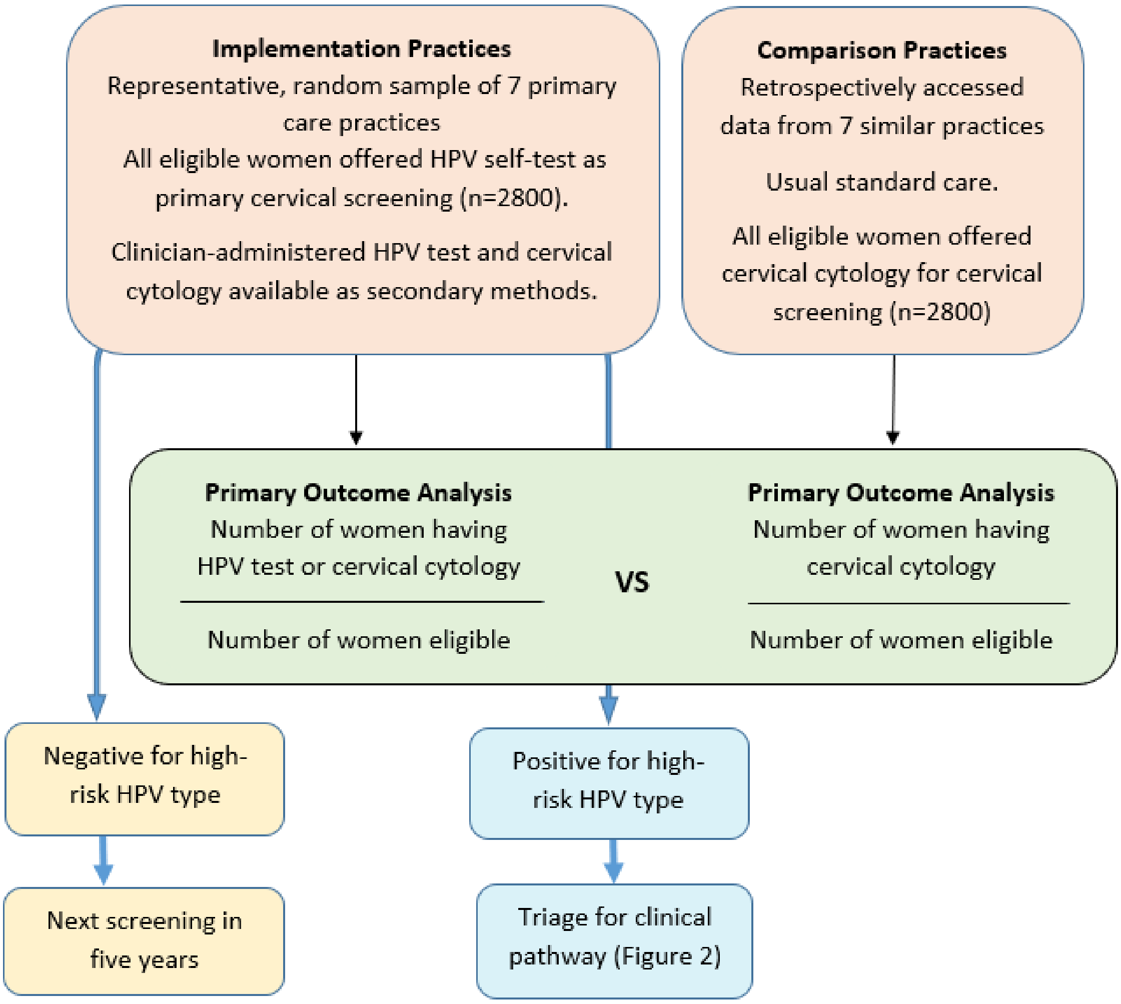


## Overview

***Cervical cancer is one of the few PREVENTABLE cancers. Screening can detect pre-cancer that can be treated, thereby preventing the development of a potentially fatal cancer.***

Cervical cancer is caused by high-risk types of Human Papillomavirus (HPV) and it is globally recognised that testing for the HPV virus, as a primary screen is more effective than cervical cytology (smear) in detecting cervical changes that may lead to cancer.[1 2] Screening in Aotearoa New Zealand utilises the less effective cervical cytology (smear) as a primary screen. Switching to HPV primary testing has the potential to reduce incident cervical cancers by 15% annually compared to the present cytology programme.[3 4]

Aotearoa has a National Cervical Screening Programme (NCSP) that was established in 1990 utilising a register and regular screening and recall systems centrally and through primary care. The current cervical screening programme has significant inequities. The government have recently announced

funding for a new cervical screening programme which will include the offer of HPV self-testing. However, this will not be rolled out until August 2023. Our study will answer important questions for the National Screening Unit (NSU) about how the new programme should be implemented including information on women who need further investigation after a positive HPV test.
Important questions remain to be answered about the *universal offer of HPV self-testing* approach before an equitable, high quality national programme of HPV primary screening for cervical cancer prevention is implemented.

*This health delivery grant (**Te Tai Tokerau project**) will address these unanswered questions with the research team working in partnership with the Primary Health Entity (PHE) Mahitahi Hauora in Te Tai Tokerau/Northland region.*

The present national screening programme (cervical cytology) has been functioning for 30 years and it is crucial that changing to an HPV primary testing programme is fully informed and achieved without loss of coverage and follow up. If this is maintained with the more effective HPV test then the programme will prevent more cancers and save more lives than the present cytology program. Increasing coverage with the HPV self-test will potentially contribute to a more equitable program.

*Universal offer of HPV self-testing*
The *universal offer of HPV self-testing* approach means all eligible wāhine will be offered an HPV self-test as the primary screening test. If the wāhine declines the self-test, she will be offered the conventional cervical cytology screen.

We do not yet know the best models for implementation of a high quality effective HPV testing program utilising *universal offer of HPV self-testing approach for all eligible women*.

Before adopting *universal offer of HPV self-testing* as the primary approach for the NCSP it must be proven that it achieves coverage results that are non-inferior (at least equal) to the present programme. Screening coverage is the proportion of eligible women screened in the previous three years. A screen is defined as either an HPV self-test, clinician taken HPV test or cervical cytology .rThe MOH target is to reach 80 percent screening coverage for all eligible women, including separately for women in Māori, Pacific, Asian and European/Other population groups.[5]

In addition, triage and referral to colposcopy after a positive high risk HPV test needs to be trialled to ensure these pathways are equitable.

**Evidence to date**
Our recent research (He Hapu Te Whare Tangata Randomised Controlled Trial (RCT), HRC17-193) has shown that the offer of an **HPV self-test** (without speculum exam) is successful in reaching under-screened or never-screened Māori women compared to the current offer of a routine cervical smear. [6] This project was delivered at the general practice level and any model scale up would need to be implemented through primary care organisations (PHOs) or their equivalent after the health and disability system reforms.

**Why choose Te Tai Tokerau/Northland region for this project**
Any proposed equitable cervical screening programme must be effective across a diverse population but it must also extend to those women that the programme has been failing to reach, particularly Māori and those in rural areas. Therefore, our model is based on implementation through primary care organisations (PHOs). Primary health organisations (PHOs) ensure the provision of essential primary health care services, mostly through general practices, to people who are enrolled with the PHO. PHOs are funded by district health boards (DHBs), who focus on the health of their population.

There are approximately 30 PHOs in New Zealand and they care for almost all New Zealanders.[7] Mahitahi Primary Health Entity is Northland's PHO formed through consolidation of the prior rural and urban PHOs. Te Tai Tokerau/Northland has a high Māori population and is a diverse rural/urban area. Our successful RCT (He Tapu Te Whare Tangata) offering HPV self-testing to under screened, primarily Māori and rural women was undertaken in Northland general practices. Hence, we have strong existing networks within this community, primary care and with the Northland DHB.

Our partnership, working with the Ministry of Health, will inform the NSU how to best implement a high quality, equitable, efficient and sustainable primary HPV screening programme. The ultimate goal is **to reduce cervical cancer incidence and death for Māori and non-Māori in Aotearoa NZ.**

**COVID 19:** Please note that since the COVID 19 emergency, screening rates have decreased sharply and the need for clarification of quality issues and implementation around the offer of *universal HPV self-testing approach* is even more urgent as this is a realistic alternative to the present system which involves an "in person" contact with an intrusive genital examination at a specified facility.

## Background

### The New Zealand National Cervical Screening Programme (NCSP*)*

The NCSP was established in 1990 to reduce the number of women who develop cervical cancer and those who die from it. Through routine screening at regular intervals, the programme aims to detect precancerous squamous cell changes, which, if not treated, may lead to cancer. Since the introduction of the NCSP in 1990 the age-standardised incidence rate of invasive cervical cancer has fallen by approximately 50% but Māori rates remain higher than those for non-Māori. Approximately 160 women are diagnosed with cervical cancer every year and 60 die from this largely preventable disease, despite the availability of an organised screening programme. [8] About half the women who develop or die from cervical cancer have never been screened, and about a third have only been screened irregularly and infrequently.[8]

### Cervical Cancer Inequities and the National Cervical Screening Programme in Aotearoa/New Zealand

Māori are more than twice as likely to be diagnosed with cervical cancer and almost three times more likely to die of cervical cancer than Pākehā. [9] [10] These cancers are preventable and disproportionately affect young Māori women. It is the second leading cause of cancer mortality for Māori women aged 25-44.[11]

**In Aotearoa (NZ) the NCSP is failing Māori women, with 35% unscreened compared to 20% of N.Z European women unscreened**[12] **and these disparities have increased due to COVID**. [13]

Cervical cancer detection programmes require high levels of screening to be successful in bringing down cancer rates.[14] The majority of cervical cancers occur in women who have either **not received screening or have had access to less frequent screens.**[15] The present cervical screening program involves a speculum exam and taking a cervical sample for cytology. Pre-cancerous cells - representing cervical intraepithelial neoplasia (CIN) - may be detected, with more abnormal cells being associated with more advanced pre-cancerous disease (often abbreviated to CIN2 or CIN3). Cytological examination may also detect carcinoma in situ or invasive cervical cancer. Abnormal results trigger a variety of follow-up pathways, which may involve colposcopic examination (microscopic examination by a gynaecologist) and biopsy.

Taking cervical samples for cytology is an invasive procedure that requires attending a clinic and has associated transport/costs. For Māori women, these are barriers to screening including having to attend a clinic, access issues due to rurality, cost, challenges with transport or time off work, fear of results, and the pain, embarrassment, (whakamā) or discomfort of a genital examination.[16]

**HPV self-testing potentially overcomes access issues for Māori and for other under-screened groups**. This project recognises that equity is for all groups. We acknowledge that it covers all peoples with cervices including trans and non-binary peoples who are underserved by the health system and will also benefit from HPV self-testing. In this study the term women is used as a surrogate for people with cervices. Hence, this proposed study examining the *universal offer of HPV self-testing* approach is important for any proposed equitable quality programme to prevent cervical cancer.

**Human Papillomavirus (HPV*)***

HPV is the causative agent of cervical cancer. Infection with certain high risk oncogenic (cancer forming) types of the HPV cause the changes in the cells of the cervix that can lead to cancer.[17] In this protocol HPV refers to these high-risk oncogenic types. While infection with HPV is common, most infections are transient and spontaneously clear without clinical consequences. However, some women develop persistent HPV infections and are at risk for cervical cancer and its precursors. HPV types 16 and 18 are responsible for 70% of cervical cancers and a group of types, (usually abbreviated to 'other') account for a much smaller percentage of cancers.[18] The knowledge about the cause of cervical cancer coupled with rapidly developing innovative preventive (vaccine) and detection (HPV testing) technologies now creates the potential to substantially reduce and eliminate the incidence of cervical cancer as called for by the World Health Organisation (WHO).[19]

The majority of New Zealand women of screening age will not be vaccinated at this stage. All women vaccinated or not, need to be involved in a cervical screening programme as the vaccine does not cover all HPV types responsible for cervical cancers.[20,21]

**Primary HPV-based screening**

HPV testing is life-saving technology. This new Evidenced Based Practice (EBP) is more effective both in detecting pre-cancer changes on the cervix, and in preventing cervical cancer than cytology.[2,21] A large body of evidence, including data from randomised trials in high and middle-income countries, has shown HPV testing as primary screening is superior to cytology in reducing cervical cancer incidence and mortality. The long term follow up of 24,510 women in the ARTISTIC RCT has now been completed and showed a similar level of protection 10 years after a negative HPV test compared with three years after a negative cytology test.[22] Analysis of four European randomised controlled trials found that, compared with cytology, HPV-based screening provided 60–70% greater protection against developing invasive cervical cancers.[2]

HPV testing in cervical screening detects high-grade lesions earlier than cytology, thus preventing more invasive cervical cancers. In addition, a negative HPV test provides greater reassurance of low pre-cancer risk than a negative cytology result and means that screening intervals can be increased to 5 years instead of the current 3 years. The Netherlands, Australia and UK have moved to HPV testing as the primary test using cervical/speculum sample in 2017, 2017 and 2019 respectively. [4,23,24] HPV testing is projected to decrease cervical cancer incidence in Australia by 15–18% and cancer mortality by 16–18%, compared with current cervical smear practice.[4]

**HPV self-test**

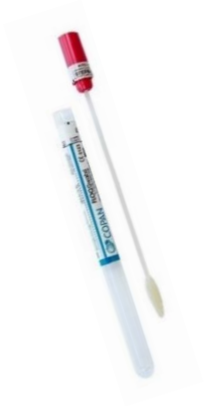


The HPV self-test, a vaginal swab, can be used for HPV screening, providing sensitivity and specificity comparable with clinician-collected specimens.[25] The test available in Aotearoa/NZ differentiates between types 16, 18 and a group of 'other" high risk types.[26 27] A no speculum, HPV self-test, has great potential to reduce disparities in screening since women are able to do the test themselves in a variety of settings including clinics, community centres and their homes. This eliminates a number of barriers for Māori women, and increases the potential to reduce disparities in detection of cervical abnormalities so reducing cervical cancer.[16] If access to the more effective innovative HPV primary testing is limited by barriers such as using the intrusive cervical/speculum sample, then there is the potential to further increase cervical cancer disparities.

**HPV self-test is acceptable and effective**

International and national studies including our recent randomised controlled trial (RCT) in Northland, suggest that HPV self-testing is acceptable and effective.[14 28 29]

The 'IMPROVE' non-inferiority trial was carried out to compare self-testing with clinician-collected testing and showed a similar accuracy of detection for high grade pre-cancer between the two groups validating this approach.[28]

Our He Tapu Te Whare Tangata randomised controlled trial (RCT) [6] offered HPV self-testing to under screened women. It successfully engaged under or never screened Māori women in both testing and follow-ups. These women had either never had a smear or not had one for more than 4 years. Results show that the offer of HPV self-test reached over 50% of under-screened Māori women compared to 21% of the control (that is women who were offered a smear). In women who had never screened, the offer of self-test reached over 40% of women compared to just 12% of never screened in the control group. More than 80% of women took their own swab and a significant number (around 20%) took the self –test in their own home. The importance of multiple access points for taking the swab and the effect of community outreach were identified as vital to the success of the study.

The results of the RCT provide a strong argument for resources to be directed to general practices to carry out this project.

**Proposed new national cervical screening programme and the Te Tai Tokerau project**

At the time of designing this study the New Zealand Ministry of Health (MOH) planned to shift the cervical screening programme to primary HPV testing in the future[30] and as we were developing the study protocol the government announced funding for a new cervical screening programme which will include the offer of HPV testing. This shift will happen in 2023. The MOH has formed an HPV primary screening project group (the Technical Reference group) to advise the programme (PI Dr Lawton is a member). As Aotearoa New Zealand adopts the *universal offer of HPV self-testing* approach, we will be the first programme in high-income countries offering self-testing as the main test. This *universal offer of HPV self-test* has the potential for significant national and international impact.

This **Te Tai Tokerau project** will inform the MOH as to the non-inferiority of the *universal offer of HPV self-testing* approach. This is necessary before any such programme can be implemented.

**The ultimate goal is the successful implementation of an effective, sustainable, and equitable cervical screening programme.**

## Methodology

**Aim**

The primary aim of this Te Tai Tokerau project is to test whether the cervical screening coverage utilising the *universal offer of HPV self- test programme* approach is non-inferior to the cervical screening coverage in the current cytology cervical screening programme.

We hypothesise that

The *universal offer of HPV self-testing* approach leads to the same or more numbers of women having a cervical screen compared to usual cervical cytology screening (includes all women due a screen, under-screened and never-screened women).

The *universal offer of HPV self-testing* programme approach is where the HPV self-test is the screen offered to all women due a cervical screen. Women can have cervical cytology as an alternative screen if they prefer this option, although the HPV self-test will be the preferred pathway in this approach.

**Study design**

We propose an implementation trial that will compare the uptake of screening at the implementation practices utilising the *universal offer of HPV self-testing* approach, to the uptake of screening from practices utilising current standard of care: cervical cytology alone.

Implementation trial

**Implementation practices**

A representative, random sample of primary care practices from the Mahitahi Primary Health Entity will be invited to participate. These practices will offer HPV-self-testing universally over a two-year period.

**Comparison practices**

Seven similar Mahitahi primary care practices (the comparative practices) who will be business as usual offering cervical cytology screens will be asked for permission to retrospectively access their screening data. De-identified data for the same period as the implementation trial will be requested.

**Data analysis**

**Primary outcome analysis**: comparing the screening coverage of two approaches.

1. Universal offer of HPV self-test approach
2. Offer of cervical cytology as per current approach

The two approaches will be compared using a Linear Mixed Model with a logit link, with practice fitted as a random effect. Non-inferiority for the primary outcome will be evaluated by observing whether the lower bound of the two-sided 95% confidence intervals for the difference in screening coverage between the two arms is above the non-inferiority limit of -10. If non-inferiority is evident, assessment as to whether the implementation has effectiveness superior to that of standard care will be carried out using the same approach but comparing to a zero difference. The primary analyses will be carried out on an intention-to-treat (ITT) basis. Per-protocol sensitivity analyses will also be conducted excluding participants who had major protocol violations. To account for any potential imbalance in underlying risk factors for screening uptake, sensitivity analyses will adjust for age, ethnicity, deprivation index, screening history and primary care practice.

**Secondary outcomes**

The following secondary outcomes will be collected from practices participating in the implementation:

1. Proportion of women having an HPV self-test
2. Proportion of women having either an HPV self-test, clinician HPV test or cervical cytology (uptake of screening)
3. Proportion of women having an HPV test with a positive result for HPV 16, 18 and or HPV 'other'.
4. Proportion of women having an HPV test with a positive result for HPV 'other' who then have a cervical cytology test (as proposed by national screening programme)
5. Proportion of women having an HPV test with a positive result for HPV 'other' and a negative/mild dysplasia cytology result who return for an HPV test after 1 year (as proposed by national screening programme)
6. Proportion of women with HPV 16, 18 attending colposcopy as per triage
7. Prevalence of high-grade lesions (CIN2, CIN3, Carcinoma) among women attending colposcopy

These outcomes will be stratified by, or adjusted for, ethnicity, deprivation index and screening history to understand screening uptake and post-screening care pathways in these groups.

**Qualitative outcomes**

8. Barriers and facilitators to the triage system for women with an HPV positive test.
9. Barriers and facilitators to delivering HPV self-testing in primary care.
10. Barriers and facilitators to women beginning screening at 25 years of age.
11. Barriers and facilitators to accessing screening, for women who are not enrolled in primary care.

These outcomes will help inform implementation of the preferred triage system for HPV positive self-tests, and also illuminate any barriers to applying this triage system, supporting an equitable triage system for positive HPV results.

**Ethics**

National ethical approval has been granted by the Southern B Health and Disability Ethics Committee (reference number 21/STH/188) with locality assessment approval by Northland DHB, Kaunihera Kaumātua and Te Poutokomanawa.

**Steering group**

In accordance with the principles under Te Tiriti o Waitangi the steering group is to work in partnership with the research partners supporting the implementation and delivery of the Te Ara Waiora Trial by providing subject matter and regional expert knowledge and following decision making processes.

Our steering group is chaired by wāhine Māori who live in Te Tai Tokerau. Membership includes:

- Research Kaumātua
- Whānau and Consumer Representative
- Mahitahi Hauora PHE Representative(s)
- Principal Investigator
- Research Project Manager
- Northland District Health Board (NDHB) Colposcopy Representatives
- Equity Lead
- Primary Screening Representative

The steering group will connect the research to communities and mitigate risk. The group will define the mode and frequency of their meetings and at present are meeting monthly.

**Internal data management committee (DMC)**
We have asked the HRC if an external data monitoring committee is required. In addition we have formed an internal data management committee. to assess data and clinical safety. The DMC will be responsible for monitoring the overall conduct of the clinical trial. Membership of the DMC includes:

- Principal investigator (general practitioner)
- Two lead investigators (gynecologists),
- Study manager
- Study statisticians
- Representatives from Mahitahi Hauora.

The DMC is chaired by an independent researcher (public health physician).

The DMC will provide recommendations about the stopping or continuing of the trial. To contribute to enhancing the integrity of the trial, the DMC may also formulate recommendations relating to the selection/recruitment/retention of participants, their management, improving implementation of the study protocol and retention of participants, and procedures for data management and quality control.

The DMC will be advisory to the steering committee. The steering committee will be responsible for promptly reviewing the DMC recommendations, to decide whether to continue or stop the trial, and to determine whether amendments to the protocol or changes to the study conduct are required.

The group will meet monthly by teleconference during the course of the study to review any issues. The research team based in the community and primary care will have immediate access by phone to one of the group members to discuss any issues as they arise. The group will report to the whole research team as necessary.

**The partnership: Mahitahi Hauora and Te Tātai Hauroa o Hine Victoria University of Wellington**
This project is a partnership between Te Tātai Hauora o Hine Victoria University of Wellington and Mahitahi Hauora. Mahitahi Hauora is Northland's Primary Health Entity (PHE) and is a collaborative endeavour with support from the Northland District Health Board, Māori led health providers, general practice, and Northland Iwi Leaders. Their goal is to support a primary healthcare system that sustains equitable, self-determined wellbeing and ensures every person has an opportunity to live a long and healthy life.[31] In their support letter for the HRC grant application Mahitahi stated:
*"Cervical cancer is a very important issue for both Māori and non-Māori in Te Tai Tokerau. Any proposed new screening programme should be acceptable and effective for our population. If we get it right here it will likely be generalisable to the rest of Aotearoa and improve access for Māori with a more effective test to reduce cervical cancer disparities."*
Mahitahi are involved at all levels, contribute and co-fund research staff, have membership on the steering committee and internal monitoring group and we will work closely with them. The Hauora will have ownership of their data and all results and publications will be approved by them. This partnership is much valued and there will many opportunities for learning from each other. The project includes local workforce development as there are named investigators from Mahitahi and they will be involved in all research outcomes as well as governance and advisory roles. Consultation for this study has occurred with GPs, PHE, community and DHB in Te Tai Tokerau region.

**Study Population: Māori and non-Māori women aged 25-69 years living in Te Tai Tokerau/Northland region**

Our study site is Te Tai Tokerau where Māori women have almost twice the incidence of cervical cancer compared to non-Māori and almost four times the mortality.[32] In Northland, cervical screening coverage for Māori is approximately 66% (Table 1).

**Table 1: NCSP coverage (%) in the three years ending 31 March 2016 by ethnicity, women aged 25–69 years, Northland District Health Board**

| Ethnicity | Population | Women screened in last 3 years | 3-year coverage | Additional screens to reach 80% target* |
|---|---|---|---|---|
| Māori | 12,680 | 8,398 | 66.2% | 1,746 |
| Pacific | 691 | 408 | 59.0% | 145 |
| Asian | 1,692 | 1,004 | 59.3% | 350 |
| European/Other | 26,874 | 21,208 | 78.9% | 291 |
| **Total** | **41,937** | **31,018** | **74.0%** | **2,532** |

**For the total population the number of additional screens is the number required to move from the total population coverage to 80%. This may not be the same as the sum of additional screens required for each ethnic group to reach 80%.*

## Inclusion criteria

The NCSP eligibility policy is that "anyone with a cervix or vagina who has ever been sexually active should be offered three-yearly cervical screening from age 25 to age 69." For this study all women aged 25.00 to 69.99 years and eligible for cervical screening to prevent cervical cancer in the year of the implementation. Includes first cervical screening test (HPV self-taken or clinician taken swab test, or cervical smear for cytology) for women aged 24.5 years and women who have a routine cervical screening test (HPV self-taken or clinician taken swab test) for cytology up to 6 months BEFORE their cervical screening test due date.[1]

Women who have symptoms raising the possibility of cervical cancer, including post-coital bleeding, unexplained intermenstrual bleeding, any post-menopausal bleeding, unexplained persistent unusual vaginal discharge or unexplained persistent deep dyspareunia should have an HPV test but the focus for these women is primary care follow-up investigation of their symptoms.

## Exclusion criteria

Women who have had treatment for cervical abnormalities and are under active follow up (e.g. still undergoing test of cure) are not eligible. A test of cure is HPV testing and cytology (co-testing) on two occasions, 12 months apart. Women who have had treatment for cervical abnormalities in the past and are overdue for test of cure are eligible.

### Sample size

The NSCP coverage report from December 2019 for the Northland DHB shows that approximately 72.5% of women (33,403/46,090) aged 25-69 were screened over a period of 3 years.[12] A total of fourteen practices and 5600 women (seven practices offering HPV-self-testing universally over a one-year period to approximately 2800 women, and seven practices providing routine clinical care

[1] HDEC amendment approved December 2021

data for approximately 2800 women about cervical cytology uptake) will give 95% power at a one-sided significance level of 2.5% to detect a non-inferiority margin of 10% between the two groups (i.e. the screening rates at participating practices will be no worse than 10% less than the screening rate obtained from the comparative data). This margin of error was chosen as we believe it is the smallest value that is clinically relevant. For this sample size we assumed a screening rate of 72.5% in the comparative data and 72.5% in the participating practices, and an average practice size of 400 participants and intra-cluster correlation coefficient of 0.01.

**Practice selection**
In total there are 28 practices from Mahitahi that are eligible (women aged 25-69 and practice is able to provide data). Of these there are 22 practices that have a moderate to high proportion of Māori (≥15% of the practices population).

To inform the implementation of the new NCSP, at the direct request of the NSU, the original HRC funded study will be broadened to include more practices and address the study aims in a shorter time. As the original study is already underway, a two-stage process of practice randomisation and selection is required.

**Stage One**
Four practices will be randomly selected from the 22 eligible practices that have a moderate to high proportion of Māori, and this random selection will be stratified by practice size and rurality. To do this the 22 practices will be divided into the following four population size by rurality groups: small-rural, small-urban, large-rural and large-urban. Within each of the population size by rurality groups, one practice will then be randomly selected using computer generated random numbers (which gives a total of four practices). The randomisation sequence will be generated by the trial statistician.
Consent to participate in the implementation will be sought from each of the four practices. If a practice does not consent, then another practice will be randomly selected that has the same population size by rurality group.

**Implementation practices:**
The four practices will provide a *universal offer of an HPV self-test* approach. Practice staff will be encouraged to invite the women to self-test. Posters and flyers with self-test HPV information will be displayed in these practices. HPV swabs and self-tests are funded by the study and there will be no charge for women. However normal service fees for the practice will apply. For example, if an eligible woman comes into the practice for a consult and is opportunistically screened she will be charged for the consultation as usual but there will be no additional charge for the HPV test.

**Comparison practices:**
Four practices (but with similar characteristics to the implementation practices will be asked retrospectively for permission to access data from records obtained through clinical care for comparison to the implementation practices. De-identified data for the same period as the implementation trial will be requested retrospectively. If a practice does not give permission to access data, another practice will be approached until four practices have given permission.

Any changes over time in this usual standard care for each practice will be documented.

**Stage Two**
To enable availability of research findings in a timely manner to inform the national screening programme the number of practices was increased to 14 practices (seven implementation

practices and seven comparison practices). This will achieve the primary aim of testing the "non-inferiority hypothesis" in 12 months. Please see the "Sample size" section for further detail.

The same methods used above in stage 1 will be used to randomly select an additional three practices for the implementation arm from the remaining eligible practices. The random selection will be stratified by practice size and rurality. Within each of the small-rural and small-urban practices, one practice will be randomly selected (n=2). The third practice will be randomly selected from the large practices. This gives a total of seven practices in the implementation arm.
An additional three practices with similar characteristics to the implementation practices will be asked for permission to retrospectively access data from records obtained through clinical care for comparison to the implementation practices.

At the completion of Year 1 the study will have reached the number of participants needed for analysis. In agreement with the Ministry of Health we will offer HPV self-testing in Year 2 to comparison clinics for up to one year or until the new national screening programme starts. Women will still be asked to complete a consent form as this will be part of the research. The research team will use canSCREEN as the database for this group to ensure clinical safety.

**Implementation practice training**
Key clinician champions identified by Mahitahi will undertake a clinical update and train the trainers programme. This will be provided by expert members of the research team. These key clinician champions will then undertake to visit each implementation practice and train clinicians and kaiāwhina and community workers. These trainers will be supported by the experts in the research team. The training will include comprehensive patient information and teaching aids, which will be available in Te Reo and English[2] with easy to follow flow charts and diagrams. Materials have been developed in consultation with the community during our previous study in this rohe, and are being re-visited for this study. Consent and information sheets are in the appendix.
One-to-two hour workshops will be held in each practice or suitable venue involving:

- Presentations on HPV, its role in cervical cancer, prevention, colposcopy
- Role-playing of real life scenarios e.g. how to get proper informed consent, how to teach women to take their own vaginal swab
- Question and answer sessions.
- The practical logistics of labelling swabs, assigning study number, transporting swabs and organising result giving

The practices will co-ordinate the testing, community outreach, results and follow up.

**Taking the swab**
Women will be given the participant information and consent form and if they agree they will be consented. This will include consent for copy of HPV results, (and if colposcopy is required, copies of histology results) to be sent to the senior researchers as well as the ordering health practitioner. Consented women will receive verbal, written and /or pictorial instructions from a doctor, nurse or kaiāwhina (community health worker) about how to perform the HPV self-test, a self-collected vaginal sample.

Women will self-collect a vaginal sample using a nylon-flocked swab (Copan Floqswab, Copan, USA). The instructions will detail how to insert the swab into the vagina and place it into a collection container.

[2] We may also translate materials into other languages if required by the practices, for example Samoan and/or Tongan

Women are at the centre of this study and we are led by their preference of where the swab will be taken. Most swabs will be taken by women in the practice, some opportunistically when the woman attends for another reason. If women feel uncomfortable about administering the swab themselves it can be taken by the clinician. Some women will do the test in their homes during a trained kaiāwhina visit and the swab returned to the practice by the kaiāwhina for routine lab collection. For some, this may involve leaving the swab in the letterbox for the kaiāwhina to pick up. While we will not encourage mailing the swab, this will be an option if required by women. All lab request forms must have a clinician's name on it as the clinician is responsible for giving HPV results (not the kaiāwhina).

**Labelling**

Every practice has labelling machines and this will done the same way as for other tests. Kaiāwhina who are making home or marae visits can take pre-printed labels with them if they know the person they are recruiting to the study. However, they may also be able to recruit other women while visiting a home or community centre if that woman is registered with the practice and due or overdue a smear. In these instances the kaiāwhina can manually write labels for the test samples and add the NHI on return to the practice. The test sample will be labelled as other tests are routinely done with the name, date of birth and unique NHI number of the woman being tested. When informed consent is obtained from the woman she will be asked how she wishes to obtain the results and the clinician/ kaiāwhina will document this. Women will also be asked if they can be contacted at a later date by one of our qualitative researchers and if so, contact details will be written on the consent form.

**Transport**

Specimens will be transported at room temperature to Southern Community Laboratories (SCL) in Dunedin who are contracted to perform the testing.

**Laboratory Test and Process**

HPV genotyping will be carried out using PCR platforms to distinguish HPV-16 and HPV-18 from other high-risk types and from negative samples. All main New Zealand laboratories have the capability to perform the recommended HPV PCR testing. The use for this study on dry vaginal swabs has been validated in international published studies and the contracted lab Southern Community Laboratories will agree to do this 'off-license' testing as part of this study.

**Results**

Results of the HPV assay test will be sent electronically encrypted (as other routine results) to the ordering primary care clinician at the primary care health practice and entered into a recall database. The database will enable recall and reminders to be sent to the woman.

**Giving results**

The primary care clinician will give the results to the woman by the method she opts for at the time informed consent is obtained. This might be by text if negative or by phone or in person at the practice. If the test is 'unsatisfactory' or 'invalid' for testing the lab will request another sample from the woman taken ideally within 6-12 weeks. Invalid results are very unusual with HPV testing.

**HPV negative result**

The woman will be informed of the result and advised that she will not need another test for at least five years. This can be an HPV self-swab as the NSU will implement HPV testing in 2023 .

**HPV high-risk positive result**

Women may have results that are positive for HPV 16, 18, or other high-risk HPV types. Results will be given to these women in person, face-to-face (kanohi-ki-te-kanohi) or by telephone (depending on

participants chosen method) in a sensitive and appropriate way. Women may choose to have whānau or friends to support them when receiving the results. It will be emphasised by the clinician that having an HPV high-risk positive results DOES NOT mean that the woman has cancer – this is very unlikely - but it may indicate that there are pre-cancer changes on the cervix. These changes can be identified and treated BEFORE they develop into cancer. Women will receive as much information as they need about the referral process to colposcopy and the colposcopy process itself using NSU information already available as well as verbal information from the trained primary care clinician. Women and whānau can be navigated through these steps. Women will be supported to colposcopy through existing support networks where available.

**canSCREEN database**

The new NCSP has funding to switch to a new primary HPV screening register in 2023. Until then, the NCSP cannot automatically register HPV results on the NSU database, as there will be no accompanying cytology. The ordering health practitioner and the research team are therefore responsible to follow up on these results.

For this project we will adapt the canSCREEN database to perform the equivalent function as the NCSP for women who have an HPV test. The canSCREEN database is a fully developed cancer screening registry platform developed and operated by VCS Foundation in Australia which will be modified to meet the needs of Te Tai Tokerau/Northland.

The canSCREEN database will hold information about HPV testing and follow-up: practice attended, HPV test results; cytology triage (for HPV-other); colposcopy referral, attendance and histology; and treatment/ discharge/ 12-month follow-up. The canSCREEN database will function as a safety net, supporting healthcare staff to identify and contact women who have not presented for follow-up tests and/ or colposcopy appointments. The canSCREEN database will also hold information relevant to monitoring eligibility and safety such as name, address and date of birth.

We anticipate at the conclusion of the project, data contained within the canSCREEN database will be migrated to the NCSP to support women's ongoing participation in cervical screening, unless individual women opt-out of this process.

The database will be modified to adapt to the needs of Northland health care providers and women in terms of how unique identification and inputs and outputs are managed within the screening pathways eg mobile interfaces, off and online, reminders and work lists can be paper based, sent by email, or SMS. Business analysts and developers will work closely with the researchers and clinicians to map the clinical pathways and requirements into the business rules of a Northland module of the register. Depending on travel restrictions and allowances between Melbourne and New Zealand, this will either be in person (preferred option) or by Zoom/Teams.

The platform is highly secure and supports role based access by which healthcare workers and research staff can only access agreed data and functions. Data are held securely on the Azure platform and hosted by VCS. Access is only available by named investigators, clinical staff and digital support staff. All VCS staff are required to comply with strict confidentiality requirements including signing annual declarations to comply with confidentiality requests.

**Triage** (see Figure 2.)

If a woman has a positive high-risk HPV result then it is the responsibility of the ordering health practitioner to organise the referral for colposcopy and the senior co-ordinating researcher or project manager will also liaise with the health practitioner to ensure clinical safety. A standardised

pro-forma letter will be sent to the colposcopy practice with usual clinical referral information stating that the woman is part of this study and why cytology is not available nor NSU registration. See below for study process at colposcopy.

Triage for colposcopy after using HPV primary screening has not been tested in Aotearoa. What is recognised is that referring all HPV positive women straight to colposcopy would increase the numbers of unnecessary colposcopies dramatically and potentially overwhelm the secondary service. To mitigate this and because the HPV 'other' types are much less likely to cause pre-cancer changes and most women will actually clear the virus in time, a triage system will use HPV testing combined with cervical cytology. The Te Ara Waiora Project working with the NDHB colposcopy team and the MOH expertise group will trial the preferred system of triage. If an HPV self-test is positive for types 16 or 18 (causing 70% of cervical cancer) the woman will be referred to colposcopy. If the test is positive for HPV 'other' types the woman will be called back for a cervical smear for cytology to triage for whether she needs colposcopy then, or can be re-tested for the HPV one year later. If the woman declines cytology after an HPV 'other' test she can have a repeat HPV test at 9 months but particularly if she is under or never screened and declines to have cytology she may be referred straight to colposcopy. If the woman does not have cytology after three months of her positive HPV 'other' result, she should be referred to coloposcopy. This will be discussed with the colposcopy team and allows for the pathway to be woman centred and more flexible.

**Figure 2: Triage**

**Figure 2. Te Ara Waiora – Implementing HPV primary testing in Aotearoa New Zealand: triage and referral pathways following an HPV test.**

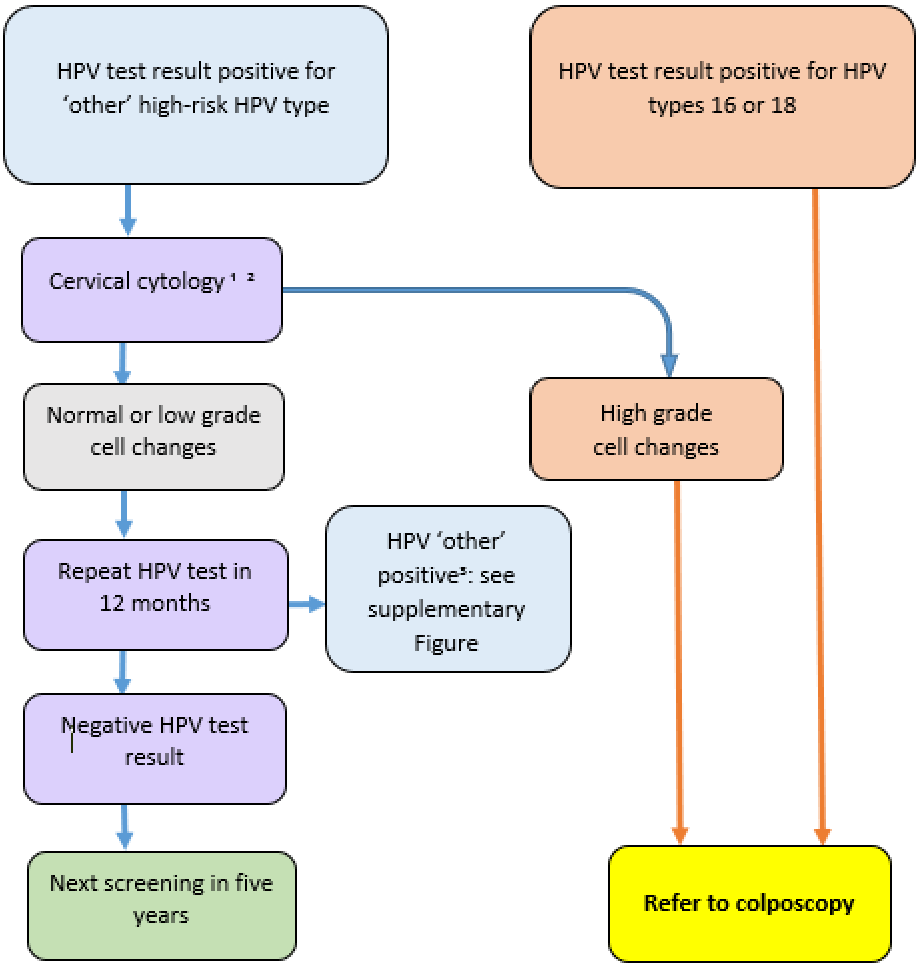


[1] Women who are under-screened or never screened and have a positive HPV 'other' high risk result but decline cytology may be referred for colposcopy or return for another HPV test in 9 months.

[2] Women who are immunosuppressed and have a positive HPV 'other' result should be offered cytology AND referred for colposcopy.

[3] Those who have a positive HPV 'other' result on their second test in 12 months AND who are over 50 should be referred to colposcopy. See supplementary Figure for detail.

**Supplemental Figure: Triage and referral pathways for women who have two HPV 'other' high-risk test results 12 months apart.**

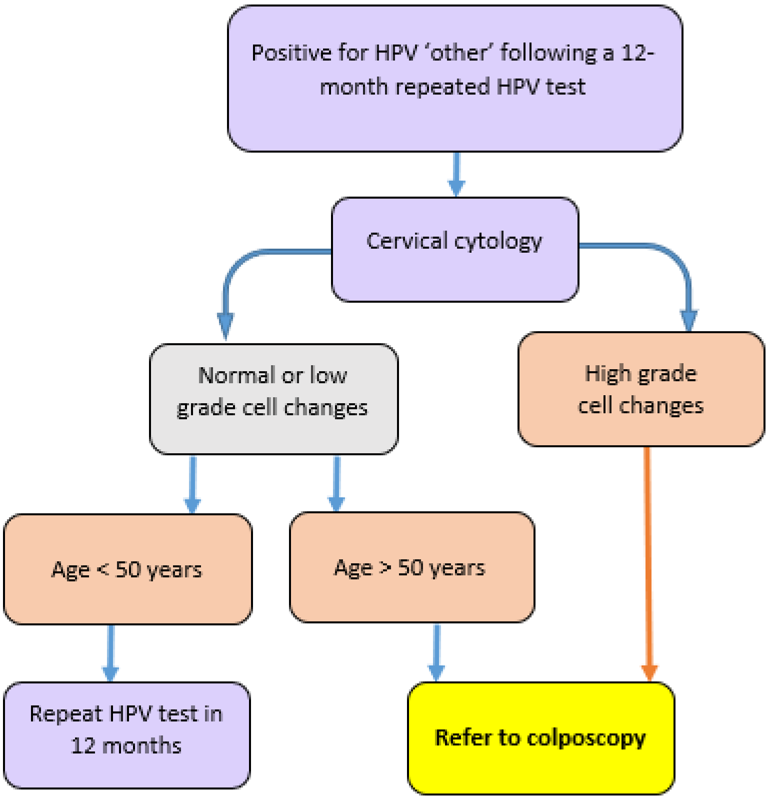


**Figure 4: Triage for women with previous abnormality, not undertaking test of cure, not back to 3 yearly cytology and/or lost to follow up**

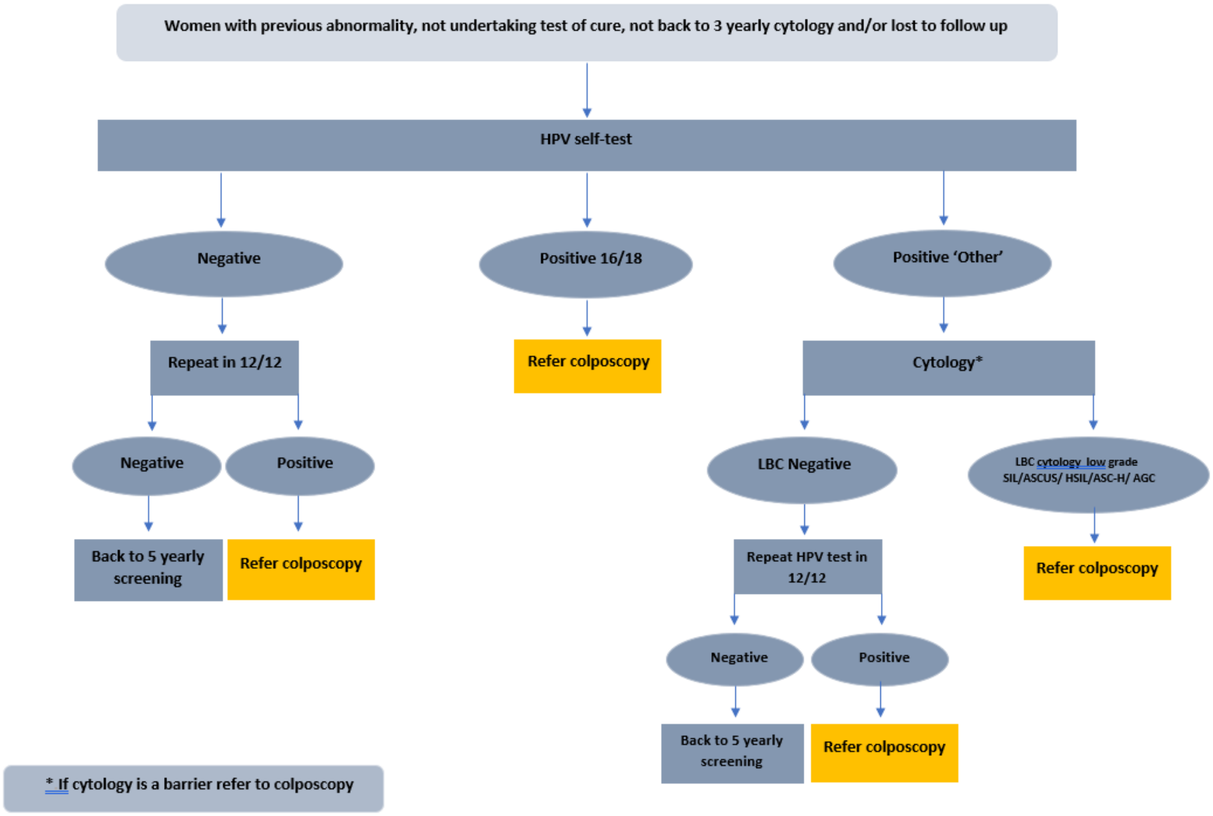


**Women with recent treated abnormality who have not yet completed TOC are excluded from HPV self-test study** [HPV and cytology annually until both negative in 2 consecutive years] **as per current NCSP guidelines**

**Barriers and facilitators to the triage system for HPV positive women**

There are unique questions about triaging that we will answer through qualitative data collection especially about women who receive a positive HPV 'other' result upon an HPV self-test who will be triaged to cytology (see Figure 2). Wāhine from the following groups will be included:

- Up to 10 women who test HPV HR16 or 18 and go straight to colposcopy
- Up to 10 women who test HPV HRother and go through the triage system
- Up to 10 women who test HPV HRother and choose to go straight to colposcopy [3]

Interviews will take place at a time and location of the participants choosing. Although the research is not restricted to Māori participants, we anticipate that many will be Māori (given the population of Te Tai Tokerau). The researchers will draw on Kaupapa Māori Research practices, which have also been described as Community-up research practices, whereby whakawhanaunga (developing relationships), participant safety, and research accountability are paramount.[33 34]
Questions include, from the women's perspectives: how does the triaging work? what barriers and facilitators exist on this new pathway? This is especially important for never/under-screened women who find current screening (cervical cytology) unacceptable. We need to ensure that triaging does not create barriers to diagnostic care.[16]
Perspectives of key stakeholders such as practice managers, doctors, nurses, kaiāwhina and PHE representatives on experiences of implementing HPV self-testing for primary care will provide critical knowledge on barriers and facilitators to implementation.

**Enablers for 25 year olds to participate in screening**
At the request of the NSU, we will also be focussing on 24/25 year old wāhine to explore what would encourage them to participate in screening, for example free screening, mail-out, self-sampling, offer of free HPV vaccination. We will address this with action research including qualitative focus groups. It is important to capture the voices of these wāhine. Multiple focus groups across different ethnicities, especially wāhine Māori and Pacific women, sexualities and abilities would strengthen this kaupapa. Our proposed action research methodology component added to the existing study will involve kaumātua, kuia and different locations including Marae and sports venues. Action research will enable 24/25 year olds to develop their research skills and will underpin the research with different experiences and worldviews of wāhine in this age group. In addition to hearing about views of free screening/mail out/self-sampling/offer of free vaccination we will also trial other methods such as direct approach to women from NGOs, kaiawhina and primary care practices.

**Support needed for participants invited via a population based register and not enrolled in primary care to participate in screening**
The NSU have also asked us to hear from whānau who are not enrolled in primary care about support needed to participate in screening. Our community partners have told us there is difficulty with transient addresses in identifying these wāhine but they can often be found with the connections, expertise and trust of kaiāwhina and kaumātua. We will aim to have focus groups or interviews with up to 20 wāhine and 10 kaiāwhina/kaumātua community workers/other primary care staff. The team is very experienced in community research and able to connect with those wāhine who the system is not readily engaging with.

**Training requirements for staff who are not registered sample takers but could be trained to deliver swabs to those wishing to take a self-sample outside of the medical clinic setting**

We will be delivering training to kaiāwhina, health coaches and health improvement practitioners for the study. We will interview these staff about training requirements and how to strengthen these roles within the system of screening, which may include micro-credentialling. Our study partners Mahitahi alerted us to the gap in knowledge around support to screening services. This includes support in accessing the HPV self-test but also the cost of supporting women to colposcopy. These contracts are held by Māori health providers in Te Tai Tokerau. We propose interviews with those who provide support to screen services to find out facilitators and inhibitors to providing this service.

**Level of knowledge and educational needs for providers**

This is an important issue, for example gaps in knowledge about how to explain screening and HPV testing to women. We will hear from a wide range of providers, with steering committee, community and PHE input and qualitative interviews. These will highlight what providers need to know to help women navigate the screening system and understand their screening results.

**What support do women need to get their results? After positive test, after negative test?**

Our previous qualitative research has highlighted the support women need to get their results and support to colposcopy. The qualitative component of our existing study answers unique questions about triaging through qualitative data collection especially about women who receive a positive HPV 'other' result upon an HPV self-test who will be triaged to cytology. We can extend these interviews to include women who have negative tests. Perspectives of key stakeholders such as practice managers, doctors, nurses, kaiāwhina and PHE representatives on experiences of implementing HPV self-testing for primary care will provide critical knowledge on barriers and facilitators to implementation.

**What we need to do to ensure women are followed up for positive HPV tests, including the level of support needed**

-Rates of follow up

-Colposcopy and follow-up cytology

- How to improve liaison between labs/primary care/colposcopy services

Our implementation study will answer these questions. We will identify questions such as who is responsible for follow-up, what is working well and why, as well as challenges to communication and information sharing. We will explore who makes colposcopy appointments and test ideas for different ways of streamlining the screening system in ways that women are kept safe. Our goal is to inform a seamless system by sharing data and regular meetings.

Data will be analysed thematically, with participants' talk grouped into themes and subthemes.[35] Barriers and facilitators of timely access to the appropriate care will be identified.

**Colposcopy**

Based on overseas figures we would expect 2.5% of women to need direct referral to colposcopy with a positive 16/18 test, with any woman with CIN 2+ disease requiring treatment. Colposcopies will be done through the Northland DHB Gynaecology outpatients as agreed with the Chief Medical Officer (Dr Mike Roberts) and the Colposcopy team at Northland DHB (lead colposcopist Dr Kristy Wolff and Alison Carlin, lead nurse) who all support this project.

At colposcopy the woman will automatically be registered on NSU database. A cytological sample should be taken. Colposcopy will be performed in the routine fashion and documentation of colposcopy will be recorded on gynae - plus database. In view of the fact that cytology will not be available at the time of colposcopy further reducing the sensitivity of colposcopy the threshold for

biopsy should be low. In the case of an abnormal transformation zone (TZ) of cervix, it is recommended that more than 1 biopsy is taken and if the TZ is normal, a random biopsy should be taken to confirm normal histology. If the TZ is not visible, the patient will be reviewed in the light of the cytology result, HPV type and other clinical details.

**Funding for HPV tests and colposcopies**

Women will pay the usual fee at their general practice for a screening consult and the HPV test will not incur any additional cost. Mahitahi will fund the HPV laboratory tests for the first stage of the study and the second stage tests will be funded by the MOH. There will be no cost to the woman for the swab or the lab test. The study will fund all colposcopies. Referral will be made straight away if the test is positive for any high risk HPV and should be seen within 20 working days. We have consulted with the colposcopy team at NDHB about the increase in colposcopies expected for the duration of the study and they have agreed to this potential increase in colposcopies and are supportive of the study.

**Funding for cytology required for HPV Other high risk**

The MOH triage process requires cytology for women with HPV Other high risk results. This will be paid for by the study.

**Data collection, extraction and management**

While Mahitahi will retain ownership of the data, the canSCREEN database will also serve as the primary source of research data relating to women who have an HPV test. The NSU is the best source of data regarding screening history and demographic information (sourced from the National Health Index). The practices will provide NHIs of participating women to the NSU to obtain information about screening history and clinical history at the start of the study time period, ethnicity, NZ Deprivation Index (socioeconomic status), and age (not date of birth). For participant safety reasons, and to be able to link to the NSU, restricted members of the research team will have access to the NHI of women in the implementation group. At the end of the implementation period, the research team will also request HPV vaccination status from the National Immunisation Register for women who participate in HPV testing. This is crucial information about expected HPV positivity and therefore provides important context for the findings related to triage, clinical care pathways and implementation.
All data will be de-identified before data analysis.

The NSU will be the primary source of research data relating to women at participating practices who opt for cervical cytology rather than an HPV test, and for comparative data from non-participating practices. For these women, the practices will provide the NHIs of the eligible population of women to the NSU (minus the women who have had an HPV test) to obtain information about cervical cytology during the implementation time period, screening history at the start of the study time period, ethnicity, NZ Deprivation Index (socioeconomic status), and age (not date of birth). The practices will remove the NHI and provide the research team with the individual line data for analysis. If there are cases where the relevant information cannot be obtained from the NSU, demographic information will be obtained from the Ministry of Health National Health Index dataset.

Data for analysis will be stored on the secure Victoria University of Wellington server, in restricted access folders and password protected files. No individuals will be identifiable in the reporting of study findings.

**Regular progress meetings**

Key CWHR and Mahitahi Hauora investigators have a regular weekly catch up and ongoing project management meetings. The wider research team already meet via zoom teleconference every two weeks. This will continue with additional monthly zoom meetings of the internal data safety-monitoring group. At other times key members of the research team can be contacted directly by the health practitioners and community workers by phone or zoom. Key members of the research team will also visit the practices at least 3 monthly throughout the study for interim updates and support.

**Proposed Timeline**

Due to COVID19 related delays and a change in leadership at Mahitahi, the first year of the project has been spent preparing for the trial (see chart below). The revised timeline means implementation will run from October/November 2021 year to October 2023. The remaining programme time will be spent analysing and disseminating results.

| Task | Oct 2020 – Oct 2021 | November 2021 – October 2022 | November 2022 – October 2023 | October 2023– Oct 2024 |
|---|---|---|---|---|
| • Ethics<br>• Mahitahi partnership team and champions assembled<br>• Train the trainers<br>• Media/ pamphlets etc<br>• Colposcopy triage confirmed locally and nationally<br>• Regular three monthly meetings with MOH, colposcopists and stakeholders<br>• Steering group and Advisory Board meetings | | | | |
| • *Universal offer of primary HPV self-testing* (clinician sampling available if patient wishes)<br>• Agreed triage occurs<br>• Data collection commenced<br>• Ongoing consultation with all stakeholders<br>• Regular progress meetings with local PHE team<br>• Advisory Board meetings | | | | |
| • Practice follow up of all women who require | | | | |

| | | | | |
|---|---|---|---|---|
| repeat HPV test in one year<br>• *Universal offer of primary HPV self-testing*<br>• Agreed triage occurs<br>• Follow up HPV tests for HPV 'other'<br>• Data collection<br>• Ongoing consultation with all stakeholders<br>• Regular progress meetings with local team<br>• Advisory Board meetings | | | | |
| • Agreed triage occurs<br>• Follow up HPV tests for HPV 'other'<br>• Analysis<br>• Advisory Board meetings<br>• External audit analysis of results<br>• Write up<br>• Dissemination of Results | | | | |

**Write up of results**

All co-investigators will contribute to the write up of the results under the lead authorship of the principal investigator.

**Dissemination of Results**

We have a dissemination plan specific to our project. Our goal is to improve outcomes and this requires our partnership (researchers, community and primary care) having ongoing consultation with multiple groups. Our priority for the dissemination of results will be firstly the community and Māori health organisations and strategic organisations that have been part of the research process. Maintaining the link between academia and Māori communities is part of our commitment to ongoing consultation and dissemination. Dissemination and consultation with the clinical workforce, health services (DHBs) and policy makers is a priority to inform about results and to bring about change.

Importantly we will have ongoing consultation with the National Screening Unit with the goal that this community implementation, if successful, will directly inform the future changes to the National Cervical Screening Programme, prioritise Māori and lead to a programme that meets the needs of Māori. Dr Lawton (PI) is on the Technical Advisory Group for the introduction of the NZ primary HPV testing programme. She is a previous member of the National Kaitiaki Group and the Māori Equity Advisory Group for the introduction of the HPV vaccine into NZ.

We have established multidisciplinary clinical networks in all 20 DHBs that are keen to be involved in receiving this knowledge and in change management activities.[36] Multiple opportunities will be taken to disseminate results at a range of Māori health and other hui such as: Māori health provider Board and staff hui, and marae committees. At a regional and central level, our focus is to achieve change

and influence policy and practice hui may include: Māori Women's Welfare League, National Kaitiaki group, workshops, educational courses, policy groups, advisory committees, Te Ora, Ngā Neehi Māori, He Hono Wahine etc. In addition, we plan to publish our findings in high ranked peer review publications and presentations at indigenous forums to share and learn.

**Conflict of interest statement**

Co-investigators are clinicians with clinical responsibility for participants. This is acknowledged by the research team as potential for conflict of interest. However, the dual role of clinician being also researcher in this study would appear to be appropriate as the participant will have her usual clinician providing care, advice and follow up. This will be fully disclosed and discussed with the participants. Co-investigators are not remunerated on the basis of recruiting participants to the study.

## References:


1. Porras C, Hildesheim A, González P, et al. Performance of self-collected cervical samples in screening for future precancer using human papillomavirus DNA testing. *JNCI: Journal of the National Cancer Institute* 2015;107(1)
2. Ronco G, Dillner J, Elfstrom KM, et al. Efficacy of HPV-based screening for prevention of invasive cervical cancer: follow-up of four European randomised controlled trials. *Lancet* 2014;383(9916):524-32. doi: 10.1016/s0140-6736(13)62218-7 [published Online First: 2013/11/07]
3. Bains I, Choi YH, Soldan K, et al. Clinical impact and cost-effectiveness of primary cytology versus human papillomavirus testing for cervical cancer screening in England. *International Journal of Gynecologic Cancer* 2019;29(4):669-75.
4. Medical Service Advisory Committee. Medical Services Advisory Committee recommendations for HPV testing Canberra 2018 [Available from: https://wiki.cancer.org.au/australia/Guidelines:Cervical_cancer/Screening/Medical_Services_Advisory_Committee_recommendations_for_HPV_testing accessed 20th April 2020.
5. National Screening Unit. Cervical screening coverage. Ministry of Health [Available from https://www.nsu.govt.nz/health-professionals/national-cervical-screening-programme/cervical-screening-coverage Accessed 16 June 2021]
6. MacDonald EJ, Geller S, Sibanda N et al. Reaching under-screened/never-screened indigenous peoples with human papilloma virus self-testing: A community-based cluster randomised controlled trial. *Aust NZ J Obstet Gynaecol* 2021; 61: 135-141 DOI: 10.1111/ajo.13285).
7. Ministry of Health. About Primary Health Organisations 2020 [Available from: https://www.health.govt.nz/our-work/primary-health-care/about-primary-health-organisations accessed 20th April 2020.]
8. National Cervical Screening Programme, Wellington: Minstry of Health; 2020 [Available from: https://www.nsu.govt.nz/health-professionals/national-cervical-screening-programme accessed 10th April 2020.
9. Ministry of Health. Ngā mana hauora tutohu: Health status indicators. Wellington 2018 [Available from: https://www.health.govt.nz/our-work/populations/Māori-health/tatau-kahukura-Māori-health-statistics/nga-mana-hauora-tutohu-health-status-indicators accessed 10th April 2020.
10. Ministry of Health. Cancer: New Registrations and Deaths 2012. Wellington2015 [Available from: https://www.health.govt.nz/publication/cancer-new-registrations-and-deaths-2012 accessed 10th April 2020.
11. Robson B PG, Cormack D. Unequal Impact: Māori and Non-Māori Cancer Statistics 1996–2001. Wellington: Ministry of Health, 2005.

12. Ministry of Health. NCSP New Zealand Total District Health Board Coverage Report: period ending 31 March 2019. Wellington Ministry of Health, 2019.
13. National Screening Unit. August 2020 [Available from: https://www.nsu.govt.nz/news/national-cervical-screening-programme-%E2%80%93-sector-update-august-2020 accessed 21 June 2021]
14. National Screening Unit Gaffney DK, Hashibe M, Kepka D, et al. Too many women are dying from cervix cancer: Problems and solutions. *Gynecologic oncology* 2018;151(3):547-54.
15. Hider P, Dempster-Rivett K, Williman J, et al. A review of cervical cancer occurrences in New Zealand 2008–2012. *NZ Med J* 2018;131:53-63.
16. Adcock A, Cram F, Lawton B, et al. Acceptability of self-taken vaginal HPV sample for cervical screening among an under-screened Indigenous population. *Australian and New Zealand Journal of Obstetrics and Gynaecology* 2019;59(2):301-07. doi: 10.1111/ajo.12933
17. Franceschi S, Denny L, Irwin KL, et al. Eurogin 2010 roadmap on cervical cancer prevention. *International journal of cancer* 2011;128(12):2765-74.
18. Chrysostomou AC, Stylianou DC, Constantinidou A, et al. Cervical Cancer Screening Programs in Europe: The Transition Towards HPV Vaccination and Population-Based HPV Testing. *Viruses* 2018;10(12):729. doi: 10.3390/v10120729
19. WHO. Draft: Global Strategy Towards The Elimination of Cervical Cancer as a  Public Health Problem 2019 [Available from: https://www.who.int/docs/default-source/documents/cervical-cancer-elimination-draft-strategy.pdf?sfvrsn=380979d6_4.
20. Smith MA, Hall M, Lew J-B, et al. Potential for HPV vaccination and primary HPV screening to reduce cervical cancer disparities: Example from New Zealand. *Vaccine* 2018;36(42):6314-24.
21. Huh WK, Ault KA, Chelmow D, et al. Use of primary high-risk human papillomavirus testing for cervical cancer screening: interim clinical guidance. *Gynecologic Oncology* 2015;136(2):178-82.
22. Gilham C, Sargent A, Kitchener HC, et al. HPV testing compared with routine cytology in cervical screening: long-term follow-up of ARTISTIC RCT. *Health Technol Assess* 2019;23(28):1-44.
23. Aitken CA, van Agt HM, Siebers AG, et al. Introduction of primary screening using high-risk HPV DNA detection in the Dutch cervical cancer screening programme: a population-based cohort study. *BMC medicine* 2019;17(1):228.
24. Public Health England. Significant landmark as primary hpv screening is offered across England London2020 [Available from: https://phescreening.blog.gov.uk/2020/01/23/significant-landmark-as-primary-hpv-screening-is-offered-across-england/ accessed 22nd April 2020].
25. Arbyn M, Verdoodt F, Snijders PJ, et al. Accuracy of human papillomavirus testing on self-collected versus clinician-collected samples: a meta-analysis. *The lancet oncology* 2014;15(2):172-83.
26. Abbott Molecular. Realtime High Risk HPV. 2018 [Available from: https://www.molecular.abbott/int/en/products/infectious-disease/realtime-high-risk-hpv accessed 20th April 2020.
27. Poljak M, Ostrbenk A. The Abbott RealTime High Risk HPV test is a clinically validated human papillomavirus assay for triage in the referral population and use in primary cervical cancer screening in women 30 years and older: a review of validation studies. *Acta dermatovenerologica Alpina, Pannonica, et Adriatica* 2013;22(2):43-7.
28. Polman NJ, de Haan Y, Veldhuijzen NJ, et al. Experience with HPV self-sampling and clinician-based sampling in women attending routine cervical screening in the Netherlands. *Preventive medicine* 2019;125:5-11.
29. Penaranda E, Molokwu J, Hernandez I, et al. Attitudes toward self-sampling for cervical cancer screening among primary care attendees living on the US-Mexico border. *Southern medical journal* 2014;107(7):426-32.

30. National Screening Unit. HPV primary screening Wellington: Ministry of Health,; 2017 [Available from: https://www.nsu.govt.nz/health-professionals/national-cervical-screening-programme/hpv-primary-screening accessed 10th April 2020.
31. Mahitahi Hauora. 2020 [Available from: https://www.mahitahihauora.co.nz/ accessed 10th April 2020].
32. Robson B PG, Simmonds S, Waa A, Faulkner R, Rameka R,. Northland District Health Board Māori Health Profile 2015. In: Te Rōpū Rangahau Hauora a Eru Pōmare, ed. Wellington: Otago University, 2015.
33. Smith LT. Decolonizing Methodologies: Research and Indigenous Peoples. London: Zed Books Ltd 2012.
34. Te Awekotuku N. He tikanga whakaaro: Research ethics in the Māori community (a discussion paper). In: Affairs) MMMoM, ed. Wellington, 1991.
35. Braun V, Clarke V. Thematic analysis. In: Cooper H, Camic PM, Long DL, et al., eds. APA handbook of research methods in psychology, Vol 2: Research designs: Quantitative, qualitative, neuropsychological, and biological. Washington: American Psychological Association, American Psychological Association, Washington, DC 2012:57-71, Chapter x, 701 Pages.
36. National Screening Unit. NSCP New Zealand all district health board coverage report- period ending 31st December 2019. In: Ministry of Health, ed. Wellington, 2019.
